# Inhibition of ROCK ameliorates pulmonary fibrosis by suppressing M2 macrophage polarisation through phosphorylation of STAT3

**DOI:** 10.1002/ctm2.1036

**Published:** 2022-09-30

**Authors:** Qingfang Li, Yuan Cheng, Zhe Zhang, Zhenfei Bi, Xuelei Ma, Yuquan Wei, Xiawei Wei

**Affiliations:** ^1^ Laboratory of Aging Research and Cancer Drug Target, State Key Laboratory of Biotherapy, National Clinical Research Center for Geriatrics, West China Hospital Sichuan University Chengdu Sichuan PR China

**Keywords:** idiopathic pulmonary fibrosis, macrophage, polarisation, radiation‐induced pulmonary fibrosis

## Abstract

**Background:**

Emerging evidence provides mechanistic insights into the pathogenesis of pulmonary fibrosis (PF), and rare anti‐PF therapeutic method has promising effect in its treatment. Rho‐associated coiled‐coil kinases (ROCK) inhibition significantly ameliorates bleomycin‐induced PF and decreases macrophage infiltration, but the mechanism remains unclear. We established bleomycin and radiation‐induced PF to identify the activity of WXWH0265, a newly designed unselective ROCK inhibitor in regulating macrophages.

**Methods:**

Bleomycin‐induced PF was induced by intratracheal instillation and radiation‐induced PF was induced by bilateral thoracic irradiation. Histopathological techniques (haematoxylin and eosin, Masson's trichrome and immunohistochemistry) and hydroxyproline were used to evaluate PF severity. Western blot, quantitative real‐time reverse transcription‐polymerase chain reaction and flow cytometry were performed to explore the underlying mechanisms. Bone marrow‐derived macrophages (BMDMs) were used to verify their therapeutic effect. Clodronate liposomes were applied to deplete macrophages and to identify the therapeutic effect of WXWH0265.

**Results:**

Therapeutic administration of ROCK inhibitor ameliorates bleomycin‐induced PF by inhibiting M2 macrophages polarisation. ROCK inhibitor showed no significant anti‐fibrotic effect in macrophages‐depleted mice. Treatment with WXWH0265 demonstrated superior protection effect in bleomycin‐induced PF compared with positive drugs. In radiation‐induced PF, ROCK inhibitor effectively ameliorated PF. Fibroblasts co‐cultured with supernatant from various M2 macrophages phenotypes revealed that M2 macrophages stimulated by interleukin‐4 promoted extracellular matrix production. Polarisation of M2 macrophages was inhibited by ROCK inhibitor treatment in vitro. The p‐signal transducer and activator of transcription 3 (STAT3) in lung tissue and BMDMs was significantly decreased in PF in vivo and vitro after treated with ROCK inhibitors.

**Conclusion:**

Inhibiting ROCK could significantly attenuate bleomycin‐ and radiation‐induced PF by regulating the macrophages polarisation via phosphorylation of STAT3. WXWH0265 is a kind of efficient unselective ROCK inhibitor in ameliorating PF. Furthermore, the results provide empirical evidence that ROCK inhibitor, WXWH0265 is a potential drug to prevent the development of PF.

## Graphical abstract

- Unselective ROCK inhibitor WXWH0265 significantly attenuated the bleomycin and radiation induced PF by inhibiting the polarization of M2 macrophages.
- The vital role of M2 macrophages was identified during pulmonary fibrosis and emphasized the importance of M2 macrophage during the fibrosis phase.
- Furthermore, the results showed that WXWH0265 regulated the polarization of M2 macrophages by mediating the phosphorylation of STAT3.

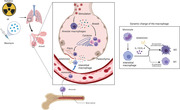

## 1 INTRODUCTION

Pulmonary fibrosis (PF) is a chronic, irreversible, pathological disease that causes a progressive decline in lung function.^1^ The main pathological features of this disease are repeated injury and repair of the lung tissue.^2^ Pirfenidone and nintedanib are two novel drugs approved by the Food and Drug Administration for the therapy of idiopathic PF (IPF).^3^ These drugs can ameliorate the lung function of patients with IPF by slowing the progression of IPF. Besides the two drugs, some new molecule inhibitors correlated to the mechanisms of PF are being revealed, but their effect is limited.^4^ Currently, there are no efficient and convenient treatment regimens for PF. Thus, anti‐PF therapeutics are urgently required.

Previous studies have shown that the Rho/Rho‐associated coiled‐coil kinases (ROCK) signalling pathway is correlated to cell proliferation, tissue repair and regeneration, and fibrocyte migration.^5^ Rho proteins have more than 20 subtypes.^6^ ROCK was the first effector protein found downstream of Rho.^7^ There are two subtypes of ROCK: ROCK1 and ROCK2.^8^, ^9^ ROCK1 is prominently expressed in the lungs, liver and kidneys, whereas ROCK2 is basically expressed in the heart and brain.^10^, ^11^ Researchers previously found that ROCK1 or ROCK2 haploinsufficiency could protect mice from bleomycin‐induced PF. Combined haploinsufficiency of ROCK1 and ROCK2 (ROCK1^+/–^2^+/–^, ROCK1^+/–^2^+/–^) provided better protection against bleomycin‐induced PF than only ROCK1^–^ or ROCK2^–^ haploinsufficiency.^12^ The Rho/ROCK signalling pathway plays an vital role in process of PF by mediating release of inflammatory factors, including tumour necrosis factor α, transforming growth factor‐β1 (TGF‐β1) and interleukin 4 (IL‐4), and influencing the immune cells polarisation.^10^ Prior researches show that the Rho/ROCK signalling pathway is closely associated with oxidative stress.^13^, ^14^ ROCK inhibitors suppress oxidative stress in hypoxia‐induced lung injury.^15^ Activated Rho/ROCK signalling also affects myosin light chain phosphorylation and regulates cell migration. The ROCK inhibitor, Y27632, reduces fibrosis in the liver and pancreas by inhibiting cell migration. Y27632 also ameliorates PF by suppressing fibrocyte migration in bleomycin‐induced PF.^16^ The Rho/Rock signalling pathway can promote the transfer of fibrocytes to myofibroblasts by generating epithelial‐mesenchymal transition (EMT), which secretes collagen. In hyperoxia‐induced PF, ROCK inhibitor suppresses the formation of myofibroblasts by reducing the production of α‐smooth muscle actin (α‐SMA).^17^ ROCK inhibitor fasudil ameliorates PF by inhibiting EMT. In bleomycin‐induced PF, fasudil reduced the production of α‐SMA in lungs.^18^ ROCK inhibitor treatment decreased the number of macrophages, but the underlying mechanism was not elucidated.^18^ In lung tissue of PF mice, the expressions of Bax and caspase‐3 were reduced by a ROCK inhibitor.^19^ Researchers also considered that the Rho/ROCK signalling pathway could be activated by radiation, but no ROCK inhibitors have previously been used in radiation‐induced PF. The impact of ROCK inhibitors in radiation‐induced PF worth to be further explore.

Various cell types in lungs facilitate the process of PF. Fibroblasts are the most prominent effector cells that transform into myofibroblasts after stimulating, resulting in large quantities of extracellular matrix (ECM) deposition in the lung alveoli and interstitium. Among immune cells, macrophages in lungs can activate myofibroblasts and play a crucial role in PF in both inflammatory and fibrotic phases.^20^, ^21^ Based on their response to stimuli, macrophages can be classified into two general groups: M1 and M2 macrophages. M1 (classically activated macrophages) promote the initial inflammatory response during the early stage of PF, whereas M2 (alternatively activated macrophages) play a vital role in tissue remodelling during the resolution phase of inflammation. IL‐4, IL‐10 and IL‐13 are crucial factors in M2 macrophages activation.^22^ These cytokines could contribute to PF by direct and indirect influences on myofibroblasts combined with or independent of the canonical profibrotic TGF‐β1.^23^, ^24^ Macrophages in various tissues are also tissue specific. Macrophages in lung include alveolar macrophages (AMs) and interstitial macrophages (IMs).^25^ The character of AMs has been extensively investigated in previous studies, but IMs have rarely been reported in PF and their role is unclear.^26^ Recent studies presented that IMs derived from monocytes in bone marrow (BM) showed a critical difference.^27^ Past researches showed that the number and proportion of macrophages in lungs of PF mice were regulated by ROCK inhibitor.^28^ However, the origin and type of these macrophages remained unknown.

WXWH0265 is a newly discovered unselective ROCK inhibitor. It has been reported that concurrently inhibiting ROCK1 and ROCK2 could significantly reduce PF as compared to independently inhibiting ROCK1 or ROCK2. So this unselective ROCK inhibitor may have marvelous anti‐fibrotic effect. To explore the effectiveness and mechanism of WXWH0265 in PF, bleomycin‐ and radiation‐induced PF models were established in this study.

## 2 METHODS

### 2.1 Mice

All mice used in the experiment were bred according to protocols approved by the Animal Care and Use Committee of Sichuan University. Inbred C57BL/6J mice were maintained in specific pathogen‐free animal colonies for subsequent experiments (Huafukang, Beijing, China).

### 2.2 Reagents and antibodies

WXWH0265 was provided by WuXi AppTec Group (Shanghai, China) and prepared in vivo in 5% dimethyl sulphoxide (DMSO) and 95% double‐distilled water. The DMSO concentration was limited to 0.1% in vitro. The signal transducer and activator of transcription 3 (STAT3) inhibitors, napabucasin (BBI608), fasudil (HY‐10341A), pirfenidone (HY‐B0673) and GSK429286A (HY‐11000), were purchased from MedChemExpress. S7936, Belumosudil (KD025) was purchased from Selleck Chemicals, USA. Clodronate liposomes were obtained from Liposomes (CP‐005‐005). Anti‐STAT3 (# 12640) and anti‐p‐STAT3 (Y705; # 9145) antibodies were purchased from Cell Signaling Technology (Cambridge, MA, USA). Anti‐α‐SMA (# MAB1420) antibodies were obtained from R&D Systems (Minneapolis, MN, USA). Anti‐ROCK1 (Cat# ab134181), anti‐ROCK2 (Cat# ab71598) and anti‐collagen‐Ⅰ (Cat# ab138492) antibodies were purchased from Abcam (Cambridge, UK). Anti‐β‐tubulin antibodies were purchased from Huabio Technology (Beijing, China).

### 2.3 Bleomycin and radiation‐induced PF

Male mice (10 weeks old) were treated with 4 mg/kg bleomycin (Selleck Chemicals), administered via intratracheal instillation, to induce PF as previously described.^29^, ^30^ Bleomycin was dissolved in 0.9% saline solution. In bleomycin‐induced PF, mice were divided into four groups: saline (control), bleomycin + saline, 10 mg/kg WXWH0265 + bleomycin and 25 mg/kg WXWH0265 + bleomycin. In radiation‐induced PF, mice were divided into four groups: control, radiation, 10 mg/kg WXWH0265 + radiation and 25 mg/kg WXWH0265 + radiation. Mice were anesthetised by intraperitoneal injection of 1% pentobarbital sodium. Whole thoraces of male C57BL/6J mice aged 10 weeks (each group have eight mice) were irradiated with 18 Gy (RS2000, 160 kV, tube current 20 mA, beam filter 0.1 mm Cu) at a dose‐rate of 3.116 Gy/min. The drug was intragastrically administered after a day of PF induction using bleomycin or radiation. Animal care and use were in accordance with the guidelines of the Institutional Animal Care and Use Committee of Sichuan University (Chengdu, Sichuan, China).

### 2.4 Hydroxyproline

Lung tissues obtained from mice were kept on ice and subjected to alkaline hydrolysis. Hydroxyproline concentration was analysed using a hydroxyproline colorimetric assay kit (Jiancheng, Nanjing, China) according to the manufacturer's instructions.

### 2.5 Histological examination

Lung samples were fixed in 10% formalin buffer (Wako Pure Chemical Industries, Ltd., Osaka, Japan) for histological examination. Fixed lung samples were sectioned (3.5 μm thick), stained with haematoxylin and eosin (H&E) and Masson's trichrome. The degree and grade of PF were determined by histological examinations and hydroxyproline levels. At least five areas (200×) of each lung of mice were assessed for severity of interstitial fibrosis by two pathologists using Szapiel^31^ and Ashcroft^32^ scoring standards. For immunohistochemistry (IHC), paraffin‐embedded pathological sections were deparaffinised with xylene and graded ethanol concentrations. Endogenous peroxide was blocked with 3% hydrogen peroxide (H_2_O_2_) for 10–15 min in the dark at room temperature. Antigen was retrieved in 10 mM sodium citrate buffer by heating in microwave for 10 min. Non‐specific binding was blocked with normal goat serum for 10 min at room temperature. Pathological sections were incubated with specific primary and secondary antibodies to detect different cells in the following sections. At least five areas (200×) from each lung of the mice were analysed using Image‐pro plus 7.0 (Media Cybernetics, USA). All images were screened using the same microscope and camera sets. The intensity of positive staining in the cytoplasm and membranes of the tissue sections was calculated as the average integrated optical density per stained area (IOD/area), as previously described with minor modifications.^33^

### 2.6 Quantitative real‐time reverse transcription‐PCR

Lung tissue and cells were analysed by a total RNA isolation kit (Foregene, Chengdu, China). Total RNA was transversely transcribed into cDNA with Prime Script RT reagent kit (Foregene). SYBR Green Real‐Time PCR master mix (Bio‐Rad, USA) was applied on quantitative real‐time reverse transcription‐polymerase chain reaction (qRT‐PCR) on CFX Connect Real‐Time PCR system (Bio‐Rad, USA). Primers used are listed in Table S1.

### 2.7 Cell culture

Bone marrow‐derived macrophages (BMDMs) were extracted from bones of the femur of C57BL/6J mice (6–8 weeks). The cells were plated in 10‐cm dishes with Dulbecco's Modified Eagle Medium (DMEM) containing 20 ng/ml of mouse Macrophage colony‐stimulating factor (M‐CSF) (R&D Systems and PeproTech) for 5–7 days. BMDMs were then stimulated with 20 ng/ml mouse IL‐4 (PeproTech), 20 ng/ml mouse IL‐10 (PeproTech), 100 ng/ml mouse Lipopolysaccharide (LPS) (Sigma–Aldrich) and 20 ng/ml mouse immunoglobulin G (IgG) (Sigma–Aldrich) in vitro, respectively.^34^ After incubation for 2 h, WXWH0265 was added to the medium. Flow cytometry and qRT‐PCR were performed after 48 h. The humidified incubator contained 95% air and 5% CO_2_ at 37°C.

### 2.8 Western blot

Western blot was performed as previously described. Approximately, 30 mg of tissue was added to 1 ml radioimmunoprecipitation assay lysis buffer with 1 mM phenylmethylsulphonyl fluoride (PMSF). Lysates were collected and centrifuged at 13 000 rpm for 10 min. Each sample contained 30 μg of protein and was mixed with buffer by heating at 95°C for 10 min. The samples were resolved on 12.5% Bis‐Tris Gel (Invitrogen Nupage Novex), transferred to a 45 μm polyvinylidene difluoride (PVDF) membrane and incubated in Tris buffered saline tween (TBST) buffer containing 5% non‐fat milk PVDF membrane, then probed with anti‐rabbit or anti‐mouse IgG/Horseradish Peroxidase (HRP) (1:10 000), after which the membranes were visualised using enhanced chemiluminescence. Western blot was performed using ImageJ software (National Institutes of Health, version 1.51k).

### 2.9 Flow cytometry

Mice were sacrificed and lung tissues obtained were minced using scissors and digested with a mixture of collagen‐Ⅰ and collagen‐IV. The suspended cells were filtered through a 70 μm nylon mesh, centrifuged at 1500 rpm for 5 min, and washed thrice with Phoshhoric acid buffer (PBS). Blood cells of the mice were directly isolated and washed three times with PBS. The cells for flow cytometry were stained with an antibody for 30 min at 4°C. Macrophages were stained with the following antibodies: Pacific Blue anti‐mouse CD11b (1:100; BD Biosciences), PE‐conjugated anti‐mouse CD206 (1:100; BD Biosciences), Allophycocyanin (APC)‐conjugated anti‐mouse F4/80 (1:100; BD Biosciences), PerCP‐CY5.5‐conjugated anti‐mouse CD45 (1:100; BD Biosciences) and Fluorescein isothiocyanate (FITC)‐conjugated anti‐mouse CD11c (1:100; BD Biosciences). Monocytes in blood were stained with the following antibodies: PE‐conjugated anti‐mouse Ly6G (1:100; BD Biosciences), APC‐conjugated anti‐mouse CD11b (1:100; BD Biosciences), PerCP‐CY5.5‐conjugated anti‐mouse CD45 (1:100; BD Biosciences) and FITC‐conjugated anti‐mouse Ly6G (1:100; BD Biosciences). The cells were analysed by flow cytometry using FlowJo software (Tree Star, Inc.).

### 2.10 Immunofluorescence

The cells were cultured in 24‐well plates, fixed in 4% paraformaldehyde, permeabilised with 0.5% Triton X‐100, and blocked with 3%–5% bovine serum albumin. The cells were incubated with anti‐p‐STAT3 and anti‐CD206 antibodies overnight at 4°C, washed three times with PBS, and incubated with fluorescent secondary antibodies for 40 min in the dark at 37°C. DAPI (4′,6‐diamidino‐2‐phenylindole) was used to stain the cell nuclei to assess cell morphology. Immunofluorescence was quantified using Image pro plus (version 6.0).

### 2.11 Primary fibroblast isolation

To isolate mouse lung fibroblasts, lungs of wild‐type mice were collected. The chest area was cleaned using 75% ethanol. Then, the lung was washed with PBS and cut into 1 cm^2^ fragments using forceps and scissors. The fragments were mixed with fibroblast medium (DMEM, 10% foetal bovin serum and 1% Non‐essential amino acid (NEAA)/L‐glutamine/Pen‐strep). Explants were then plated on 10 cm dishes in fibroblast medium for 5–7 days.

### 2.12 Lung function measurement

Mice were anesthetised with 200 mg/kg tribromoethanol and tracheotomised using a mouse ventilator (eSpira; EMMS, Edinburgh, UK). The inspiratory capacity, compliance, tidal volume and total lung capacity were measured.

### 2.13 Macrophage depletion

Clodronate liposomes (200 μl) were administered by tail vein injection on days 1, 8, 15 and 22 (control liposomes were used as a control). On day 28, the lungs of mice were isolated for flow cytometry and pathological analysis.

### 2.14 Statistical analysis

All data are presented as the mean ± standard error of the mean (SEM). Statistical analysis was calculated using the GraphPad Prism 7.0 software (GraphPad, San Diego, CA, USA). One‐way analysis of variance was performed for statistical comparison with one variable. Data are demonstrated as the mean ± SEM. All statistical analyses were performed using the GraphPad software. *p* < .05 was considered as significant.

## 3 RESULTS

### 3.1 WXWH0265 decreased the lung weight, lung index and hydroxyproline levels of bleomycin‐induced mice

**FIGURE 1:**
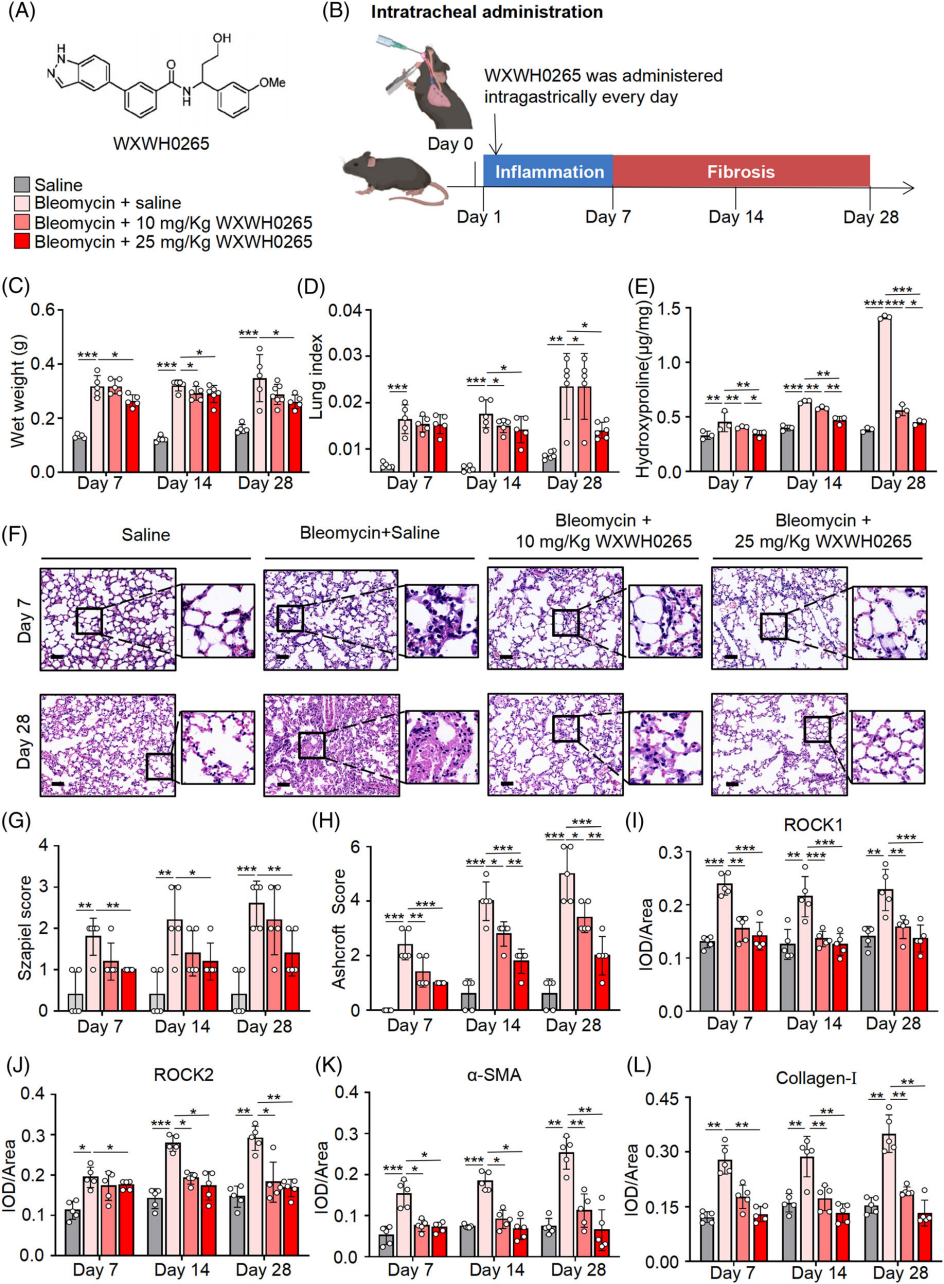
Inhibition of ROCK ameliorated bleomycin‐induced lung fibrosis. (A) The key structural features of the ROCK inhibitor (WXWH0265). (B) Schematic representation of the experimental protocol for bleomycin exposure, treatment and endpoints in bleomycin‐induced lung fibrosis mice. For bleomycin‐induced mice, 4 mg/kg bleomycin was administered by intratracheal instillation. At the day after bleomycin injection, the ROCK inhibitor WXWH0265 at 10 and 25 mg/kg was administered intragastrically every day. (C–E) After intratracheal instillation at 4 mg/kg bleomycin or saline, the mice were treated with WXWH0265 or saline. The wet weight of lung (C), lung index (D) and hydroxyproline assay in lung (E) on days 7, 14 and 28 are shown. Lung index was referred to lung/body weight ratio. (F) H&E staining of representative mice lung tissues on days 7 and 28. Representative pictures were shown, scale bar = 50 μm. (G) The inflammation changes in the lungs were quantified by a numerical inflammation score (Szapiel score) histopathological. (H) The fibrotic changes in the lungs were quantified by a numerical fibrotic score (Ashcroft score) histopathological. (I–L) The mice were sacrificed on days 7, 14 and 28 and the lung specimens were harvested for immunohistochemical analysis and stained with ROCK1, ROCK2, α‐SMA and collagen‐Ⅰ. The mean optical density of ROCK1 (I), ROCK2 (J), α‐SMA (K) and collagen‐Ⅰ (L), expression in the lung tissue on the days 7, 14 and 28. Data are shown as mean ± SEM. ^*^ *p* < .05, ^**^ *p* < .01, ^***^ *p* < .001

The main goal in this study is to explore the role of ROCK inhibitor, WXWH0265, in PF development. The structure of WXWH0265 is presented in Figure 1A. The effect of WXWH0265 on bleomycin‐induced PF was primarily evaluated based on the lung weight, lung index and hydroxyproline content (Figure 1B). Lung weight and lung index are used to evaluate the fibrosis of lung. Hydroxyproline content in lung could be used to assess the ECM deposition. Mice were treated with WXWH0265 by intragastric administration every day after bleomycin injection and then euthanised on days 7, 14 and 28 for the analysis of fibrosis. The wet weight of lungs in bleomycin‐induced PF group was significantly higher than that in saline‐treated group (Figure 1C). The wet weight of lungs in high‐dose WXWH0265‐treated group was lower than that of the bleomycin‐induced PF group at three time points. The lung index was calculated by dividing the wet weight of lung by the weight of mice. The lung index in bleomycin‐induced PF group mice was significantly higher than that in control (saline‐treated group) (Figure 1D). In WXWH0265‐treated groups, the lung index of the low‐dose (10 mg/kg) and high‐dose (25 mg/kg) groups was significantly reduced as compared with the bleomycin injection group at days 14 and 28. We further measured the hydroxyproline content in lung to determine the accumulation of ECM at three time points (Figure 1E). In bleomycin‐induced PF group, the hydroxyproline content of lung was significantly higher than that in control (Figure 1). In WXWH0265‐treated groups, hydroxyproline levels was lower than those in the group with bleomycin‐induced PF. Hydroxyproline levels were significantly lower in high‐dose group than those in low‐dose group. These data demonstrated that ROCK inhibitor WXWH0265 could ameliorate PF in general. To further identify the results, we performed the pathological section to evaluate the lung histology macroscopically and microscopically.

### 3.2 WXWH0265 alleviated the pathological changes associated with bleomycin‐induced PF in vivo

Tissue injury and ECM deposition in lung tissue caused by bleomycin were assessed by H&E and Masson's trichrome staining, respectively. Histological analysis of the H&E and Masson's trichrome staining lung sections was used as a primary assessment to evaluate the fibrotic response in mice treated with bleomycin and WXWH0265. In saline group, no obvious inflammation or fibrosis was observed in the lung tissue. Histological changes on days 7 and 28 are shown in Figure 1F.

After intratracheal injection of bleomycin, the structure of the alveoli was disordered and the airway wall was significantly thickened, in which collagen was deposited (Figure 1F). Collagen expression in bleomycin‐induced PF group was significantly higher than that in saline‐treated group after day 7, and increased progressively on days 14 and 28. There was obvious alveolar inflammation in lung tissue and leucocyte infiltration in alveoli septum on day 7. On day 14, alveolar inflammation was more severe than before, and the alveolar space was thickened. Fibroblasts and matrix accumulated in lung tissue. The number of fibroblasts and degree of matrix deposition were higher in the lung tissue on day 28. On day 28, ECM deposition in bleomycin‐induced PF group was the most severe. In WXWH0265‐treated groups, collagen deposition was low. The effect of WXWH0265 on bleomycin‐induced inflammation and PF was examined on days 7, 14 and 28 after bleomycin treatment. Although fibrotic lesions were observed in the WXWH0265‐treated groups, the extent and intensity of the lesions were lower than those in bleomycin‐induced PF group. Lung sections from WXWH0265‐treated groups presented moderate inflammatory cells infiltration, relatively normal alveolar structure, slightly thickened alveolar walls, and few lymphocytes and plasma cells. No significant morphological differences were observed between the two WXWH0265‐treated groups either. Then, Ashcroft score and Szapiel score were applied to standardised quantified the PF pathologically.

### 3.3 Histological evaluation of bleomycin‐induced PF treated by WXWH0265

The grade of alveolitis and inflammation was evaluated using Szapiel score (Figure 1G). WXWH0265 treatment at 10 and 25 mg/kg attenuated lung inflammation. The severity of PF was systematically assessed using Ashcroft score (Figure 1H). The Ashcroft score of bleomycin‐induced PF mice was significantly increased compared with saline‐treated mice. The administration of unselective ROCK inhibitor WXWH0265 resulted in a decrease in Ashcroft score.

### 3.4 WXWH0265 reduced the expression of ROCK1, ROCK2, α‐SMA and collagen‐Ⅰ in lung tissue of bleomycin‐induced PF mice

To evaluate the expression of ROCK1 and ROCK2, IHC was performed on the lung sections. The increased expression of ROCK1 and ROCK2 in bleomycin‐induced PF mice was significantly decreased by high dose of WXWH0265 (Figures 1I,J and S1). Expression of α‐SMA is considered a typical biomarker of the transformation of fibroblast to myofibroblasts, which can be observed in the alveolar and interstitial spaces of the lung. Based on IHC results, α‐SMA expression in bleomycin‐induced PF mice was significantly higher than that in saline‐treated group, and there was no significant difference between the two groups on days 7, 14 and 28 (Figures 1K and S1). In WXWH0265‐treated groups, the expression of α‐SMA in both dose groups demonstrated a remarkable decrease compared to that in bleomycin‐induced PF group. Between the two WXWH0265‐treated groups, there was no significant difference. Collagen‐Ⅰ is another vital factor for measuring ECM deposition in lung mesenchyme. In pathological sections on day 7, there was obvious collagen‐Ⅰ deposition in alveolar and interstitial spaces of the lungs (Figure 1L). Collagen‐I expression was significantly decreased in low‐dose WXWH0265 group. Collectively, the outcomes indicated that inhibiting ROCK could reduce the α‐SMA and collagen‐Ⅰ production to inhibit PF.

### 3.5 Therapeutic efficacy comparison of WXWH0265, fasudil, pirfenidone, KD025 and GSK429286A in bleomycin‐induced PF mice

To compare the effect of WXWH0265 on PF, we applied fasudil, pirfenidone, KD025, and GSK429286A as positive controls in bleomycin‐induced PF mice. Fasudil is an unselective ROCK inhibitor that is effective in the treatment of bleomycin‐induced PF at a dose of 100 mg/kg administered orally.^18^ Pirfenidone is a new drug administered to treat IPF, and its dose in mice is 300 mg/kg by oral administration.^35^ KD025 is a selective ROCK2 inhibitor, which was administered by intraperitoneal injection at 100 mg/kg.^36^, ^37^ GSK429286A is a selective ROCK1 inhibitor that has been used to reduce mean arterial pressure at 30 mg/kg in a dose‐dependent manner. Therefore, we applied GSK429286A at 30 mg/kg in bleomycin‐induced PF mice. All the drugs were administered once a day, 1 day after bleomycin treatment.^38^ The wet weight of lungs, lung index, H&E staining, Masson's trichrome staining and pathological score were used to evaluate the therapeutic efficacy of these drugs (Figure S2). Fasudil and pirfenidone significantly reduced lung wet weight and lung index (Figure S2A,B). KD025 and GSK429286A slightly decreased the lung wet weight and lung index without a statistical difference.

The grade of alveolitis and inflammation was evaluated using the Szapiel score (Figure S2C). All small‐molecule inhibitors attenuated lung inflammation. The severity of PF was systematically assessed using the Ashcroft score (Figure S2D). The Ashcroft score of bleomycin‐induced PF mice was significantly higher than that of saline‐treated mice. Administration of small‐molecule inhibitors resulted in a decrease in Ashcroft score. The results of H&E and Masson's trichrome staining are presented in Figure S2E,F. The results showed that 10 mg/kg of WXWH0265 treatment significantly decreased the degree of PF in a dose‐dependent manner, achieving equipotent therapeutic effects in the positive control with fasudil, pirfenidone, KD025 and GSK429286A. Alveolar inflammation and collagen deposition were significantly reduced after WXWH0265 treatment at 25 mg/kg. In addition, pulmonary function was applied to evaluate therapeutic efficacy, including inspiratory capacity (Figure S2G), compliance (Figure S2H), tidal volume (Figure S2I) and total lung capacity (Figure S2J). The pulmonary function significantly declined after bleomycin treatment. Wet weight of lungs, Ashcroft score and lung function were more preserved with 25 mg/kg WXWH0265 treatment compared with KD025, GSK429286A and pirfenidone treatment. Treatment with 25 mg/kg WXWH0265 or 100 mg/kg fasudil did not yield statistical significance across the anti‐fibrotic parameters. High‐dose WXWH0265 could promote pulmonary function better than other inhibitors suggesting that WXWH0265 is a potential drug for attenuating fibrotic lesions and provides a new method for PF treatment.

### 3.6 Evaluation of toxicity induced by WXWH0265 in bleomycin‐induced PF

The weights of mice were used to evaluate adverse effects (Figure S3A). Bleomycin exposure caused a significant decrease in body weight, whereas treatment with WXWH0265, fasudil, pirfenidone, KD025 or GSK429286A did not induce further weight loss. Histological examination of vital organs using H&E staining after 28 days of treatment showed no significant differences (Figure S3B). The levels of albumin, alanine transarninase, alkaline phosphatase, aspartate aminotransferase, total bilirubin, cholesterol, low‐density lipoprotein cholesterol, high‐density lipoprotein cholesterol and lactate dehydrogenase were measured to evaluate toxicity (Figure S4). However, no significant toxicity was observed. The results revealed that WXWH0265 is a novel, safe and effective drug in PF, which is a potential strategy.

### 3.7 WXWH0265 impacted the expression of classically and alternatively activated macrophage relative markers in lung tissue in bleomycin‐induced PF

**FIGURE 2:**
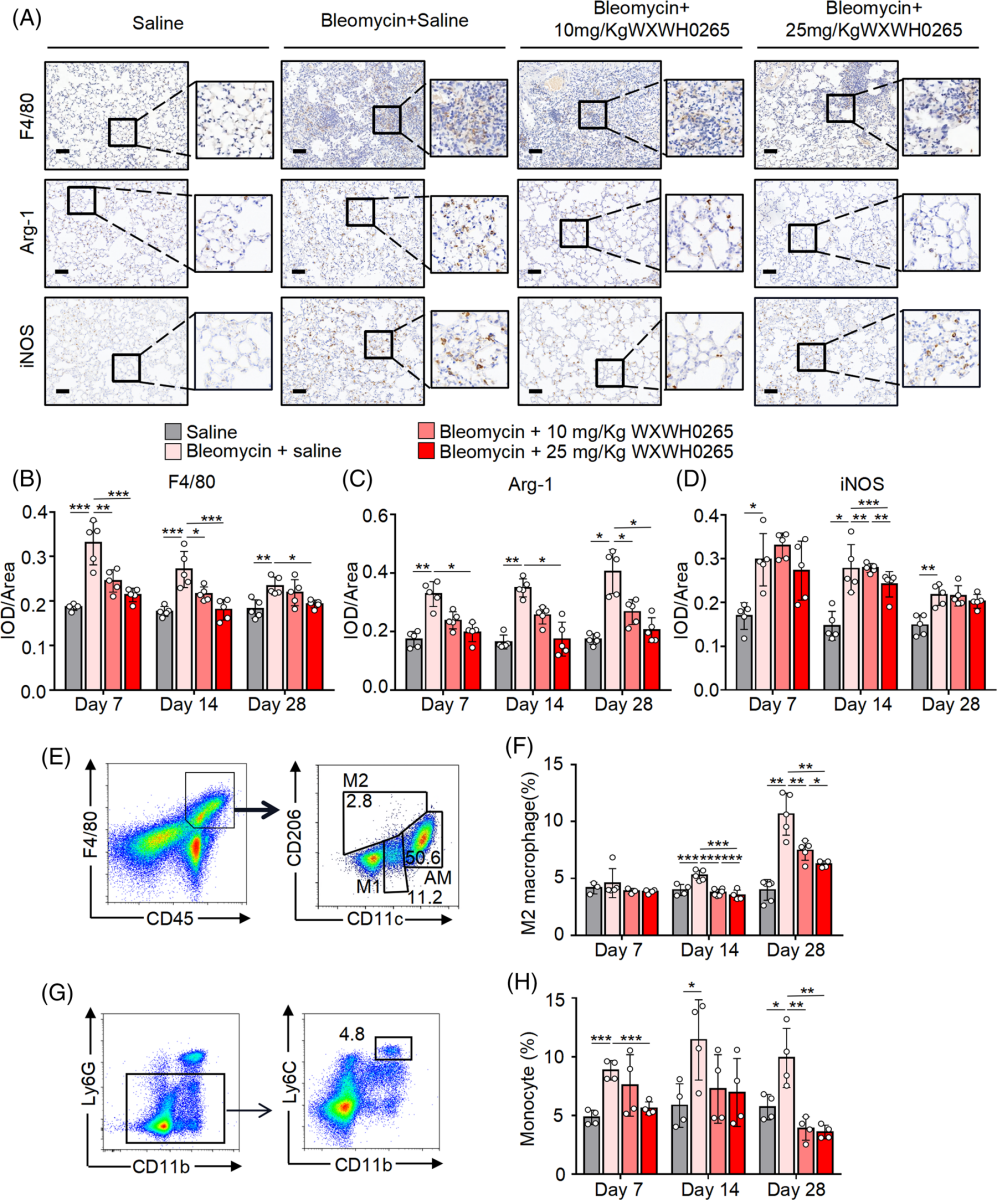
Blockade of ROCK inhibited the M2 macrophages infiltration in bleomycin‐induced fibrotic mice. Mice with intratracheal administration of bleomycin (4 mg/kg) or saline were treated with WXWH0265 (10 or 25 mg/kg) or saline. The mice were sacrificed on days 7, 14 and 28 and the lung specimens were harvested for immunohistochemical analysis and stained with F4/80, Arg‐1 and iNOS. (A) Representative pictures of IHC analysis on day 28 were shown. Scale bar = 50 μm. (B–D) The mean optical density of F4/80 (B), Arg‐1 (C) and iNOS (D) expression in the lung tissue on the days 7, 14 and 28. After the establishment of bleomycin‐induced PF, mice were treated with WXWH0265. On days 7, 14 and 28, the lung tissues and blood were collected and digested for further analyses. (E–H) The infiltration of M2 macrophages (CD45^+^ F4/80^+^ CD206^+^ CD11c^–^) in lung tissue and monocytes (CD45^+^ CD11b^+^ Ly6G^–^ Ly6C^high^) in blood was detected by flow cytometry. Flow cytometric cytometry analysis of the percentage of infiltrated M2 macrophages (E and F) and monocytes (G and H) were shown. Flow cytometry dot plots of macrophage, M1/M2 macrophage and alveolar macrophage in lung tissue of the saline‐treated mice on day 7. Data are shown as mean ± SEM. ^*^ *p* < .05, ^**^ *p* < .01, ^***^ *p* < .001

Macrophages in lungs are crucial immune sentinels that promote lung homeostasis.^39^, ^40^ After stimulation, the macrophages in lungs significantly increased causing inflammation and fibrosis.^41^, ^42^ F4/80 is a specific and precise marker of inflammation in macrophages; therefore, it can be used to evaluate macrophage levels. Immunohistochemical staining of F4/80 in lung tissue demonstrated that F4/80 levels were significantly upregulated on days 7, 14 and 28 after stimulation, with the increase on day 7 being the most significant (Figure 2A,B). The reduction in F4/80 after WXWH0265 treatment demonstrated a dose‐dependent trend. Based on their response to stimuli, macrophages can be classified into two general groups: M1 and M2 macrophages. In previous studies, the proportion of M2 macrophages was upregulated in bleomycin‐induced PF in mice.^43^ Arg‐1 is one of the most important markers of M2 macrophages, which could reflect their number and proportion. In addition, M1 macrophages play a crucial role in PF. iNOS is a specific marker of M1 macrophages, which could reflect their number and proportion. In bleomycin‐induced PF mice, the expression of Arg‐1 significantly increased at three time points compared to that in saline‐treated mice (Figure 2A,C). A high dose of WXWH0265 decreased the expression of Arg‐1 on day 7. In bleomycin‐induced PF mice, the expression of iNOS significantly increased (Figure 2A,D). On day 14, iNOS expression was downregulated by WXWH0265. At the other two timpoints, no significant difference of iNOS expression was observed after WXWH0265 treatment.

### 3.8 WXWH0265 inhibited the proportion of M2 macrophages polarisation and monocytes in blood to alleviate PF in bleomycin‐treated mice

It has been reported that AMs have superior anti‐microbial function, while IMs are equipped with more pronounced immunomodulatory capacity.^44^ AMs are primarily found in alveolar space, while IMs mainly exist in pulmonary interstitium near the alveolar septa, bronchus and vessels. The special location of IMs confers greater immunomodulatory effects during PF. In addition, IMs in interstitium of lung are closely connected with ECM, immune cells and fibroblasts, thus influencing fibro deposition.^45^ Hence, in this study, we focused on IMs to explore their role in PF using flow cytometry. Previous studies suggest that CD45^+^ F4/80^+^ CD206^+^ CD11c^+^ cells in mouse lung microenvironment could be considered as AMs. The number of M2 macrophages is relatively higher than that of M1 macrophages after stimulation.^46^ CD45^+^ F4/80^+^ CD206^+^ CD11c^–^ cells are considered as M2 macrophages and CD45^+^ F4/80^+^ CD206^–^ CD11c^–^ cells are considered as M1 macrophages, derived from BMDMs. On day 7, the proportion of M2 macrophages in the bleomycin‐induced PF group did not increase significantly (Figure 2E,F), but on day 14, the proportion of M2 macrophages demonstrate significant increase (Figure 2E,F) as compared to the saline‐treated group (control). The proportion and absolute number of M1 macrophages did not decrease significantly after WXWH0265 treatment (Figure S5A,D). The proportion of M2 macrophages gradually increased as PF aggravated, and the absolute number decreased after WXWH0265 treatment (Figure S5C). After bleomycin injection, the proportion of AMs was not significantly changed, but the absolute number of AMs significantly increased on days 7 and 28. In addition, the ROCK inhibitor did not significantly eliminate these cells at any time point (Figure S5B,E).

After intratracheal bleomycin injection, PF processes were divided into two stages: early inflammatory and late fibrotic. The infiltration of monocytes in blood usually increases significantly after inflammatory stimuli.^47^ They play an important role in the development of PF. IM in lung tissue are derived from monocytes. Monocytes from blood were marked as CD45^+^ CD11b^+^ Ly6G^–^ Ly6C^high^. Monocytes were detected by flow cytometry on days 7, 14, and 28 (Figure 2G,H). The proportion of monocytes in blood of the bleomycin‐induced PF mice increased to twice of that in saline‐treated group on days 7 and 14, whereas the proportion decreased on day 28. WXWH0265 treatment reduced the proportion of monocytes, which was similar to that in saline‐treated group.

Macrophages can reflect inflammation in lung tissue. The expression of F4/80 suggested that inflammation in lung tissues was more severe on day 7 than on days 14 and 28 after bleomycin treatment. The roles of M2 macrophages are diverse. According to the IHC and flow cytometry results, the increase in M2 macrophages in fibrotic phase on day 28 revealed that M2 macrophages may play a crucial role in fibrosis. The application of ROCK inhibitor reduced M2 macrophages, contributing to the alleviation of bleomycin‐induced PF, which verified this hypothesis. ROCK inhibitor ameliorates bleomycin‐induced PF by regulating M2 macrophages in a dose‐dependent manner in vivo. Roles and phenotypes of M2 macrophages were explored in subsequent experiments.

### 3.9 Clodronate liposomes deplete macrophages and WXWH0265 attenuates PF via macrophages

**FIGURE 3:**
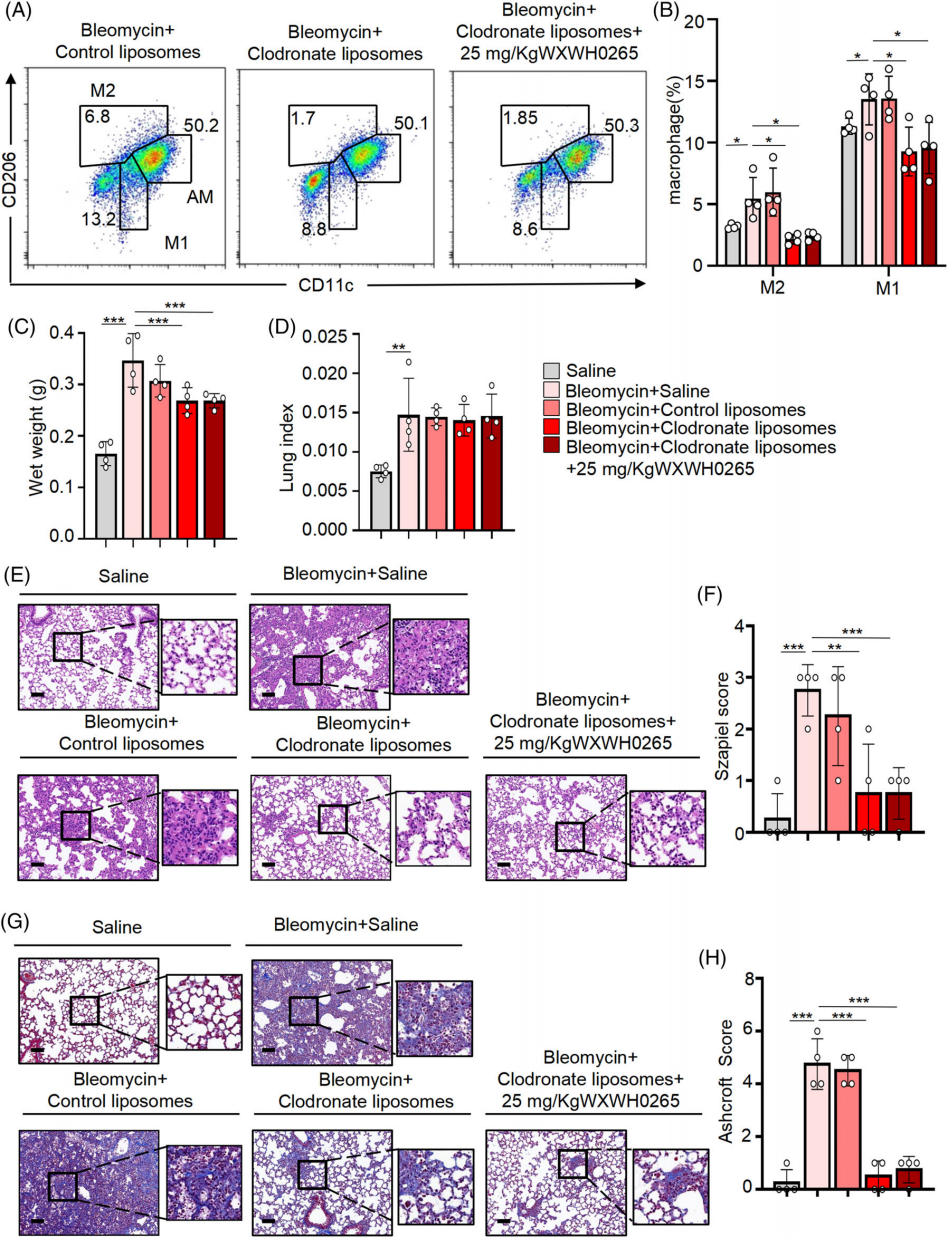
Clodronate liposomes depletes macrophages and WXWH0265 attenuates lung fibrosis via macrophages. C57Bl/6 mice were intratracheally administrated by 4 mg/kg bleomycin on Day 0 ± clodronate liposomes (200 μl) via tail vein on days 1, 7, 14 and 21. Control liposomes was given as a control. (A and B) The infiltration of M2 macrophages (CD45^+^ F4/80^+^ CD206^+^ CD11c^–^) and M1 macrophages (CD45^+^ F4/80^+^ CD206^–^ CD11c^–^) in lung on day 28 was detected by flow cytometry. Flow cytometry analysis of the percentage of infiltrated M2 and M1 macrophages. The wet weight (C) and lung index (D) was shown on day 28. (E) H&E staining of representative mice lung tissues on day 28. (F) The inflammation changes in the lungs were quantified by a numerical inflammation score (Szapiel score) histopathological. (G) Masson's staining of representative mice lung tissues on day 28. (H) The fibrotic changes in the lungs were quantified by a numerical fibrotic score (Ashcroft score) histopathological. Representative pictures were shown, scale bar = 50 μm. Data are shown as mean ± SEM. ^*^ *p* < .05, ^**^ *p* < .01, ^***^ *p* < .001

The use of clodronate liposomes is an effective strategy for depleting macrophages in vivo.^48^ The peak effect of clodronate liposomes occurred 24 h after injection and lasted up to 5 days or longer.^49^ To evaluate the effect of ROCK on macrophages derived from monocytes in PF, we applied ROCK inhibitor, WXWH0265 in mice injected with clodronate liposomes. Control liposomes was given as a control. Each bleomycin‐induced PF mice was injected with 200 μl liposomal clodronate via tail vein.^50^ Some mice were treated with WXWH0265 to observe its combined therapeutic effect on PF. Mice were sacrificed at fibrosis phages, day 28 after bleomycin treatment. The administration of clodronate liposomes significantly decreased the numbers of M1 and M2 macrophages (Figure 3A,B), but the number of AMs was not significantly reduced. WXWH0265 had no inhibitory effects on AMs (Figure S6A,B). After clodronate liposome treatment, the wet weight of lungs in bleomycin‐induced PF mice was not significantly increased, whereas the control liposomes had no protective effect (Figure 3C,D). The combination of clodronate liposomes and WXWH0265 did not reduce the wet weight of lungs or lung index. Clodronate liposome treatment significantly reduced inflammation and collagen deposition (Figure 3E,G). Inflammation and fibrotic scores showed similar trends (Figure 3F,H). Clodronate liposomes and WXWH0265 combination treatment showed no significant therapeutic effect compared to clodronate liposome treatment after bleomycin exposure. The therapeutic effect of WXWH0265 treatment declined with the decline in macrophages. Without macrophages, WXWH0265 had slight effect on inhibiting PF. Considering that WXWH0265 treatment decreased M2 macrophages in bleomycin‐induced PF, it supposed that inhibition ROCK can inhibit M2 macrophage polarisation to ameliorate PF.

### 3.10 M2 polarisation can induce myofibroblast activation and ECM production ex vivo

M2 macrophages can be further classified into M2a, M2b and M2c subtypes based on their stimulated response in vitro.^43^ M2a macrophages induced by IL‐4 or IL‐13 promote type II immune responses and secretion of profibrotic factors. M2c‐type polarisation elicited by LPS and IgG can facilitate the immunoregulation of M2c macrophages. In addition, M2c‐type polarisation stimulated by IL‐10 was associated with tissue remodelling and enhanced anti‐inflammatory responses. Moreover, M2d macrophages represent a new subtype of macrophages identified in tumours, and are known as tumour‐associated macrophages.^51^ Each M2 macrophage subpopulation has a distinct function, and specific cytokine‐secretomes and surface markers. CD206, Arg‐1, FIZZ‐1, YM‐1, CCL24, CCL1 and CXCL13 could be used to distinct their phenotypes among M2 macrophages in vitro.

**FIGURE 4:**
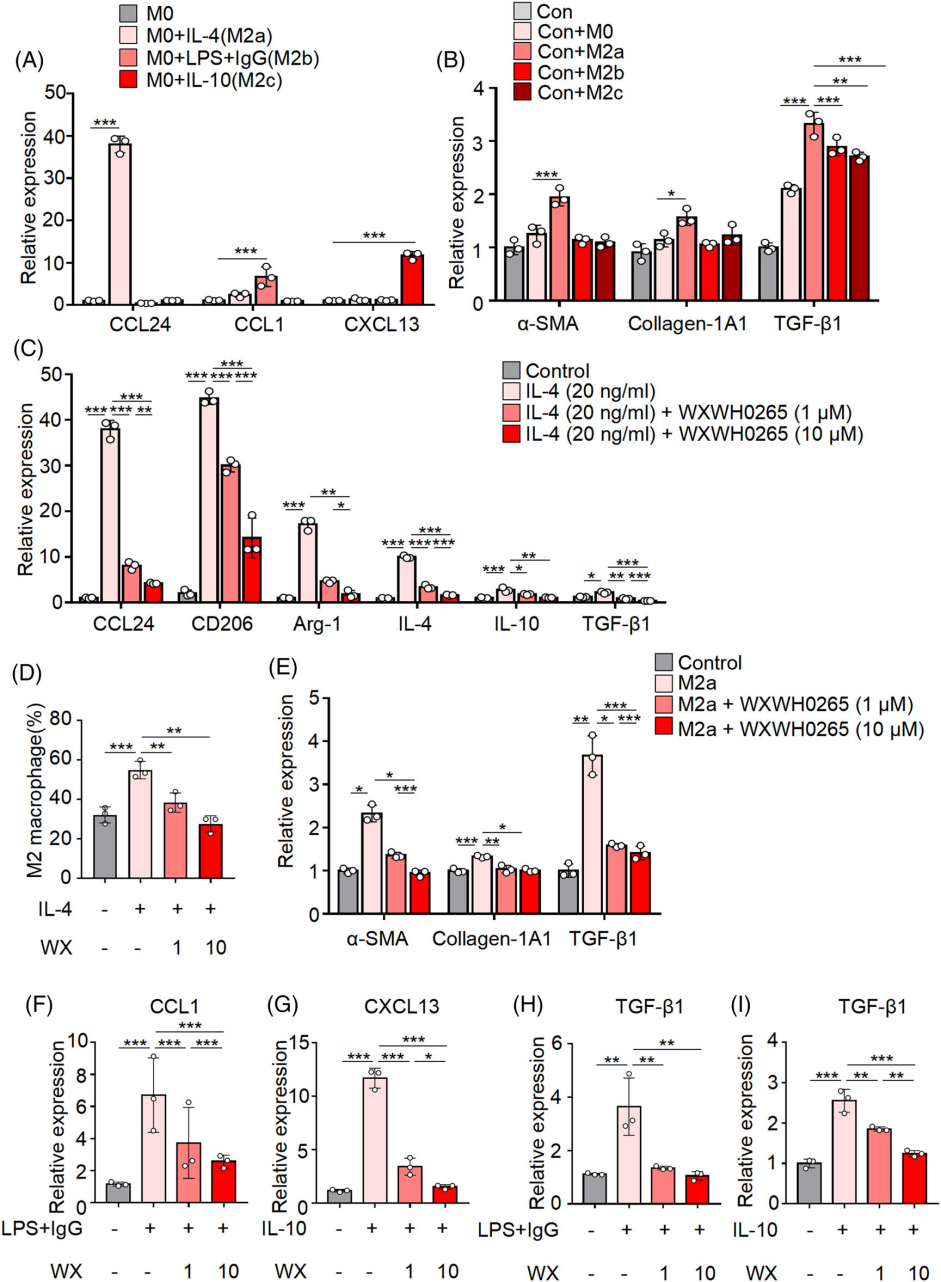
Conditioned medium (CM) collected from BMDMs‐treated lung fibroblast. BMDMs were extracted from the femurs of untreated wild‐type mice treated by M‐CSF and various cytokines in the culture medium. M2 macrophages could be further classified into M2a, M2b and M2c subtypes based on their stimulated response and BMDMs treated with M‐CSF acted as control. BMDMs were stimulated with IL‐4, LPS plus IgG, and IL‐10, respectively. The relative mRNA expressions of CCL24, CCL1 and CXCL13 were quantified by qRT‐PCR. (A) After the macrophages were stimulated for 48 h, the CM from M2a, M2b and M2c was collected. To identified which phenotypes of macrophages could stimulate the fibroblast activation, the CM from M0, M2a, M2b and M2c were used to co‐cultured with the fibroblasts, fibroblasts cultured with CM from M0 macrophages were considered as control. Relative mRNA expressions of α‐SMA, collagen‐1A1 and TGF‐β1 in the lung fibroblasts were quantified by qRT‐PCR (B). (C and D) BMDMs were extracted from the femurs of untreated wild‐type mice treated by M‐CSF and IL‐4 was added at 20 ng/ml in the culture medium. After cultured for 2 h, 10 and 1 μM of WXWH0265 was added into the medium for another 48 h and then the BMDMs were harvested for further analyses. The relative mRNA expressions of CCL24, CD206, Arg‐1, IL‐4, IL‐10 and TGF‐β1 (C) in the BMDM were quantified by qRT‐PCR. The proportion of M2 macrophage (CD45^+^ CD11b^+^ F4/80^+^ CD206^+^) was analysed by flow cytometry (D). WX: WXWH0265; the CM from M2a macrophages treated or untreated with WXWH0265 were co‐cultured with fibroblasts. The relative mRNA expressions of α‐SMA, collagen‐1A1 and TGF‐β1 in the lung fibroblasts were quantified by qRT‐PCR (E). BMDMs treated by M‐CSF and LPS together with IgG was added to the culture medium. After cultured for 2 h, 10 and 1 μM of WXWH0265 was added into the medium for another 48 h and then the BMDMs were harvested for further analyses. The relative mRNA expressions of CCL1 (F) in the BMDM were quantified by qRT‐PCR. BMDMs treated by M‐CSF and IL‐10 was added to the culture medium. After cultured for 2 h, 10 and 1 μM of WXWH0265 was added into the medium for another 48 h and then the BMDMs were harvested for further analyses. The relative mRNA expressions of CXCL13 (G) in the BMDM were quantified by qRT‐PCR. The CM from M2b or M2c macrophages treated or untreated with WXWH0265 were co‐cultured with fibroblasts. The relative mRNA expressions of TGF‐β1 in the lung fibroblasts were quantified by qRT‐PCR (H and I). Data were presented as mean ± SEM of three separated experiments. ^*^ *p* < .05, ^**^ *p* < .01, ^***^ *p* < .001

To explore which phenotypes of macrophages could stimulate the fibroblast activation and secret profibrotic markers, we co‐cultured fibroblasts with various M2 phenotype cells’ CM in vitro. After being stimulated by M‐CSF, BMDMs were untreated or exposed to various cytokines for 24 h. BMDMs were cultured in vitro and stimulated with IL‐4, LPS plus IgG, or IL‐10 to polarise them into M2a, M2b and M2c subtypes, respectively. M0 macrophages (untreated) were used as control. The transcription levels of CCL24 in IL‐4‐treated BMDMs were upregulated compared with M0 macrophages and other phenotypes, suggesting that M2a macrophages could be stimulated by IL‐4 (Figure 4A).^52^ BMDMs treated with LPS combined with IgG showed increased CCL1 expression (Figure 4A). IL‐10 activated M2c macrophages showed significantly higher levels of CXCL13 expression (Figure 4A).^53^

To identify which macrophage phenotypes could stimulate fibroblast activation, conditioned medium (CM) from M0, M2a, M2b and M2c were co‐cultured with fibroblasts. Fibroblasts cultured with CM collected from M0 macrophages were used as controls. After being stimulated by M‐CSF, BMDMs were untreated or exposed to various cytokines for 24 h. The medium was then changed to serum‐free medium, and the CM was harvested 48 h later. After 48 h, the expression levels of α‐SMA, collagen‐1A1 and TGF‐β1 were tested (Figure 4B). The expression of α‐SMA and collagen‐1A1 in fibroblasts was significantly increased with M2a CM stimulation while compared with that in other groups. When it comes to TGF‐β1, which is critical to induce PF and promote the ECM production,^54^ CM from M2a, M2b and M2c could all elevate the expression level after incubation, suggesting the comprehensive roles of the three subtypes of M2 macrophages in the fibrosis induction.

Therefore, to further investigate how WXWH0265 inhibit macrophage polarisation towards different M2 subtypes, next set of experiments were performed. Firstly, we investigated whether WXWH0265 treatment could inhibit the polarisation of M2a macrophage in vitro. M2a polarisation could be inhibited by WXWH0265 for the downregulation of CCL24, CD206, Arg‐1, IL‐4, IL‐10 and TGF‐β1 after co‐incubation with macrophages detected in the current study (Figure 4C). The flow cytometry demonstrated similar results (Figure 4D).

Moreover, addition of WXWH0265 in M2a macrophage induction by IL‐4 also depleted the activation of myofibroblasts by M2a macrophage CM (Figure 4E). In addition, although M2b and M2c macrophages suggested minor effects on α‐SMA and collagen‐1A1 generation (Figure 4B), we also studied whether WXWH0265 could inhibit M2b and M2c macrophages polarisation. Interestingly, after WXWH0265 treatment on M2b and M2c macrophages in vitro, the CCL1 and CXCL13 were downregulated with dose dependent manner (Figure 4F,G). The CM from WXWH0265‐treated M2b or M2c macrophages were co‐cultured with fibroblasts, respectively. The transcription levels of TGF‐β1 in both groups were reduced after WXWH0265 treatment, suggesting the broad inhibitory effects of WXWH0265 on M2 macrophage polarisation (Figure 4H,I).

The results of in vivo study also correlated with that in vitro study, however, the subtypes of M2 macrophages in vivo are complex. After PF induction by bleomycin, the expression of CD206, Arg‐1, FIZZ‐1, YM‐1, CCL17, CCL24, CCL1, CXCL13 and CXCR4 were upregulated, indicating that M2a, M2b and M2c subtypes may be activated (Figure S7A–I). Moreover, WXWH0265 treatment inhibited the production of CD206, Arg‐1, FIZZ‐1, YM‐1, CCL17, CCL24, CXCL13 and CXCR4 in a concentration‐dependent manner. This results also suggested that WXWH0265 might broadly affect the macrophage polarisation and its inhibitory effect of fibrosis might be a comprehensive one concerned with M2a, M2b and M2c macrophages as detected in vitro experiments.

### 3.11 WXWH0265 regulates matrix metalloproteinases in bleomycin‐induced PF mice

Profibrotic mediators can be secreted and produced by macrophages. Matrix metalloproteinases (MMPs) represent one of the most important profibrotic mediators secreted by macrophages.^55^ MMPs and tissue inhibitors of metalloproteinases (TIMPs) play a vital role in the maintenance of ECM in lung, and dysregulation of MMPs and TIMPs promotes the accumulation of ECM.^56^ We investigated whether the protective effects of WXWH0265 against lung fibrosis induced by radiation and bleomycin were correlated with the balance between MMPs and TIMPs in the chronic phase of PF. The transcriptional levels of MMP3 (Figure S7J), MMP8 (Figure S7K), MMP9 (Figure S7L), MMP13 (Figure S7M) and TIMP1 (Figure S7N) were analysed. After 28 days of intratracheal administration of bleomycin, the relative mRNA expression levels of MMP3, MMP8, MMP9, MMP13 and TIMP1 significantly upregulated in lung tissue of mice. WXWH0265 significantly decreased the expression of MMP3, MMP8, MMP9, MMP13 and TIMP1 in a dose‐dependent manner. The results demonstrated that WXWH0265 reduced mRNA expressions of MMP3, MMP8, MMP9, MMP13 and TIMP1 in lung tissue, which resulted in reduced deposition of collagen in the ECM.

### 3.12 WXWH0265 ameliorated PF in radiation‐induced mice

**FIGURE 5:**
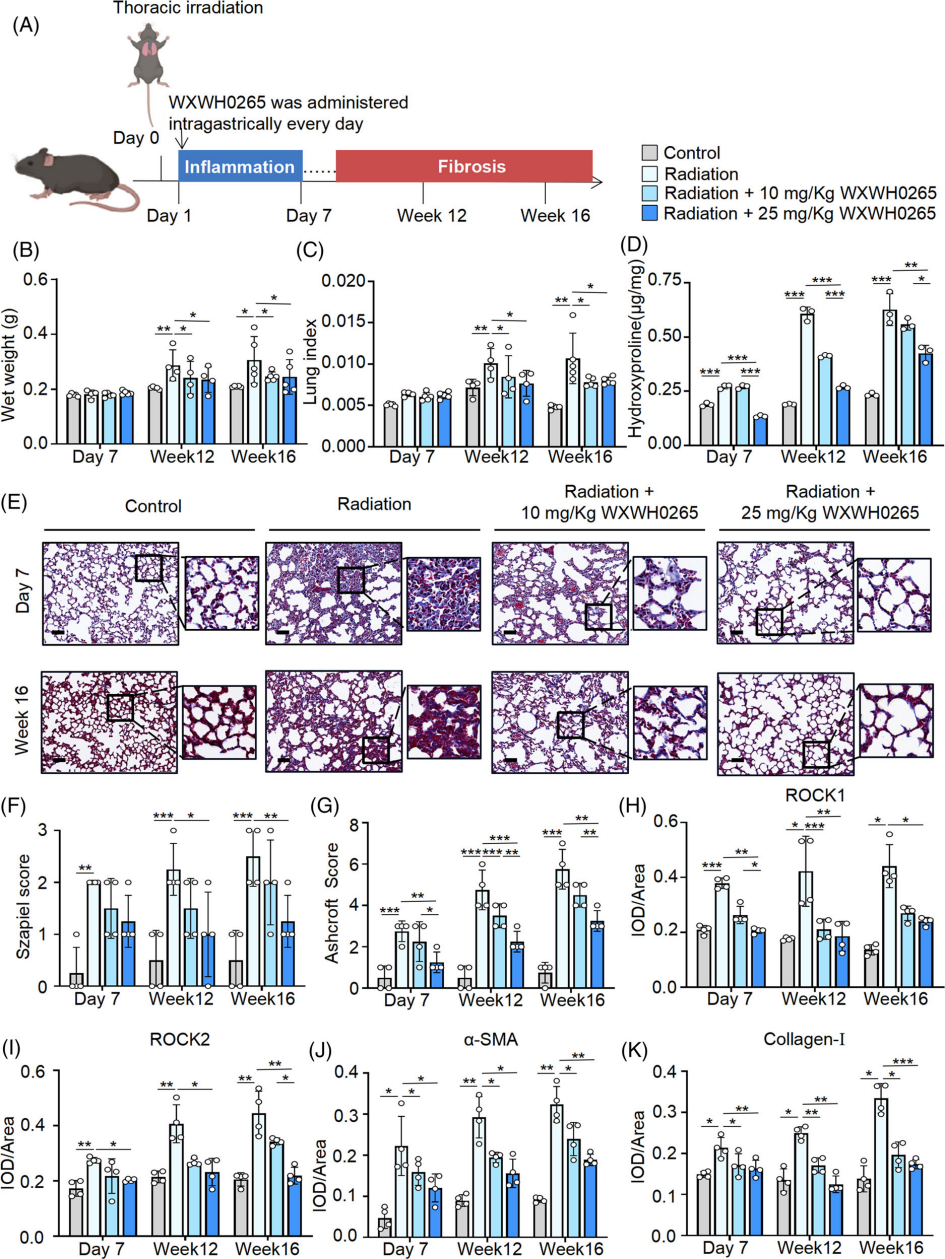
Inhibition of ROCK ameliorated radiation‐induced lung fibrosis. (A) Schematic representation of the experimental protocol for radiation‐induced lung fibrosis, treatment and endpoints in radiation‐induced lung fibrosis mice. For radiation‐induced pulmonary fibrosis, mice were exposed to a single dose of 18 Gy irradiation on the bilateral thorax. At the day after radiation, the ROCK inhibitor WXWH0265 was given at 10 and 25 mg/kg via administration intragastrical every day. At the endpoint, the lung tissue and blood were collected for further experiments. (B–D) The wet weight of lung (B), lung index (C) and hydroxyproline assay in lung (D) on day 7, week 12, week 16 are shown. Lung index was referred to lung/body weight ratio. (E) Masson's staining of representative mice lung tissues on day 7 and week 16. Representative pictures were shown, scale bar = 50 μm. (F) The inflammation changes in the lungs were quantified with a numerical inflammation score (Szapiel score) histopathological. (G) The fibrotic changes in the lungs were quantified with a numerical fibrotic score (Ashcroft score) histopathological. (H–K) The mice were sacrificed on day 7, week 12 and week 16 and the lung specimens were harvested for immunohistochemical analysis and stained with ROCK1, ROCK2, α‐SMA and collagen‐Ⅰ. The mean optical density of ROCK1 (H), ROCK2 (I), α‐SMA (J) and collagen‐Ⅰ (K), expression in the lung tissue on the day 7, week 12 and week 16. Data are shown as mean ± SEM. ^*^ *p* < .05, ^**^ *p* < .01, ^***^ *p* < .001

Previous studies have recognised the activation of ROCK and the vital role of M2 macrophages in radiation‐induced PF; however, the effect of ROCK inhibitors has not been identified.^57^ Considering the effect and safety of WXWH0265 in bleomycin‐induced PF, we further used the unselective inhibitor, WXWH0265 to treat radiation‐induced PF. In radiation‐induced mice, the changes in hydroxyproline content, lung weight and lung index were similar to those of bleomycin‐induced PF (Figure 5A–D). Histological analysis of H&E in the radiation‐treated group showed significant inflammation injury, necrosis and pneumonitis on day 7, whereas Masson's staining showed no obvious blue fibre deposition (Figure 5E). At weeks 12 and 16, extensive collagen was accumulated in radiation‐induced PF group. In WXWH0265‐treated groups, inflammation and blue fibre deposition decreased.

### 3.13 Histological evaluation of radiation‐induced PF treated with WXWH0265

Szapiel score was used to assessed the grade of alveolitis and inflammation (Figure 5F) and Ashcroft score was applied to systematically assess the severity of lung fibrosis (Figure 5G). Szapiel score showed a lower degree of inflammation in high‐dose ROCK inhibitor‐treated PF group than in the radiation‐induced PF group at weeks 12 and 16. On day 7, ROCK inhibitor failed to protect lung tissue from severe inflammation. Notably, compared to control mice, radiation‐induced PF mice had higher Ashcroft scores. Administration of the unselective ROCK inhibitor WXWH0265 led to a reduce in Ashcroft score.

### 3.14 WXWH0265 reduced the expression levels of ROCK1, ROCK2, α‐SMA and collagen‐Ⅰ in lung tissues of mice with radiation‐induced PF

The expression levels of ROCK1 and ROCK2 increased in radiation‐induced PF group and significantly decreased in high‐dose WXWH0265 treatment group (Figure 5H,I). In radiation‐induced PF mice, after treatment with low‐dose WXWH0265, α‐SMA expression did not decrease at early phase (Figure 5J). At weeks 12 and 16, WXWH0265 significantly inhibited the α‐SMA production. The change in expression level of collagen‐Ⅰ in mice with radiation‐induced PF is presented in Figure 5K. As shown in Figure 5K, collagen‐Ⅰ in radiation‐induced PF group was significantly elevated compared to control group, and collagen‐Ⅰ was decreased in a dose‐dependent manner after ROCK inhibitor treatment at three time points. Collagen‐Ⅰ content was slightly decreased in high‐dose ROCK inhibitor‐treated mice.

### 3.15 WXWH0265 influenced the expression of classically and alternatively activated macrophage relative markers in lung tissue impacted with radiation

After inducing PF via radiation, the number of macrophages in lungs significantly increased, and the increase in the number of macrophages was associated with inflammation and fibrosis.^58^ F4/80 in immunohistochemical staining indicated that F4/80 levels significantly increased on day 7, week 12, and week 16 after stimulation (Figure S8A,B). Regarding inflammation, the levels of F4/80 on day 7 were higher than those at the other two time points. Unlike bleomycin‐treated mice, the expression of Arg‐1 in radiation‐treated mice was significantly downregulated in high‐dose WXWH0265‐treated group, while low‐dose WXWH0265 did not reduce Arg‐1 expression on day 7 and week 12 (Figure S8A,C). After radiation, iNOS expression was upregulated. WXWH0265 did not decrease iNOS expression (Figure S8A,D).

In radiation‐induced PF mice, the proportion of M2 macrophages was significantly increased on day 7, week 12 and week 16. The polarisation of M2 macrophages was significantly reduced in both doses of WXWH0265, except for day 7 of low‐dose WXWH0265 treatment (Figure S8E). The proportion of monocytes in blood was detected using flow cytometry on day 7 (Figure S6F). The number of monocytes in the blood of radiation‐induced PF mice increased to three times that of control group on day 7. At week 16, the proportion of inflammatory monocytes significantly reduced. The proportion of monocytes was significantly downregulated by high dose of WXWH0265. The dynamic changes in M2 macrophages and monocytes reflect the transition from acute injury to chronic fibrosis. These results demonstrated that the changes in M1, M2 and AM were not consistent with those in bleomycin‐induced PF. Seven days after radiation, the proportion of M1 macrophages increased in inflammation phase and decreased with the progression of fibrosis (Figure S9A).

Similar to that in bleomycin‐induced PF, the expression level of F4/80 in radiation‐induced PF on day 7 was the highest among the three time points, indicating the severe inflammation in early‐stage of radiation‐induced PF. Although the change in M2 macrophages was similar, the course of radiation‐induced PF was longer. ROCK inhibitor ameliorated radiation‐induced PF by regulating M2 macrophages in a dose‐dependent manner in vivo, which indicated that M2 macrophages promote radiation‐induced PF and ROCK inhibitor is efficient in its treatment. It is essential to explore how ROCK regulates M2 macrophage polarisation.

Similar to bleomycin‐induced PF, the proportion of M2 macrophages significantly increased after radiation in the fibrosis phase. Thus, the expression levels of CD206, Arg‐1, FIZZ‐1, YM‐1, CCL17, CCL24, CCL1, CXCL13 and CXCR4 were evaluated at week 16. The expression levels of CD206, Arg‐1, FIZZ‐1, YM‐1, CCL17, CCL24, CCL1, CXCL13 and CXCR4 were elevated, demonstrating that the M2a, M2b and M2c phenotype macrophages may be activated after radiation (Figure S10A–I). All the markers were decreased after WXWH0265 treatment, which indicated that M2 macrophages were activated in radiation‐induced PF and that the polarisation of M2 macrophages could be inhibited by WXWH0265 treatment. The microenvironment of radiation‐induced PF was complex and the response persisted for a longer period than that of bleomycin‐induced PF. The phenotypes of M2 macrophages require further exploration.

### 3.16 WXWH0265 regulates MMPs in radiation‐induced PF mice

Abnormal ECM accumulation and remodelling are the hallmarks of radiation‐induced PF.^59^ MMPs and TIMPs are vital profibrotic markers secreted by macrophages, and their distribution contributes to collagen deposition. Sixteen weeks after thoracic irradiation, the expression levels of MMP3 (Figure S10J), MMP8 (Figure S10K), MMP9 (Figure S10L), MMP13 (Figure S10M) and TIMP1 (Figure S10N) were significantly upregulated. After treatment with WXWH0265, the expression levels of MMP3, MMP8, MMP9, MMP13 and TIMP1 were lower than those in the radiation‐treated PF group.

### 3.17 Phosphorylation of STAT3 acted as a regulator of M2 macrophage polarisation in vitro

STAT3 are vital transcriptional regulators in cells that participate in many pathological processes.^60^ Previous studies have demonstrated that STAT3 plays an important role in M2 macrophage differentiation.^61^ However, the role of STAT3 in PF remains unclear. Studies have demonstrated that STAT3 phosphorylation is responsible for the transcription of IL‐4 and IL‐10 genes. IL‐4 and IL‐10 are important cytokines in promoting M2 macrophages polarisation. Fostering STAT3 phosphorylation facilitates the release of IL‐4 and IL‐10.^62^ TGF‐β1 act as a profibrotic factor to promote fibrosis pathogenic routes, which is widely researched in PF. M2 macrophages are major contribution of the secretion and production of TGF‐β1. TGF‐β1 accelerates the transfer of fibroblasts into myofibroblasts to induce PF. In this section, we further explore the role of STAT3 in M2 polarisation and the secretion of IL‐4, IL‐10 and TGF‐β1 during PF.

**FIGURE 6:**
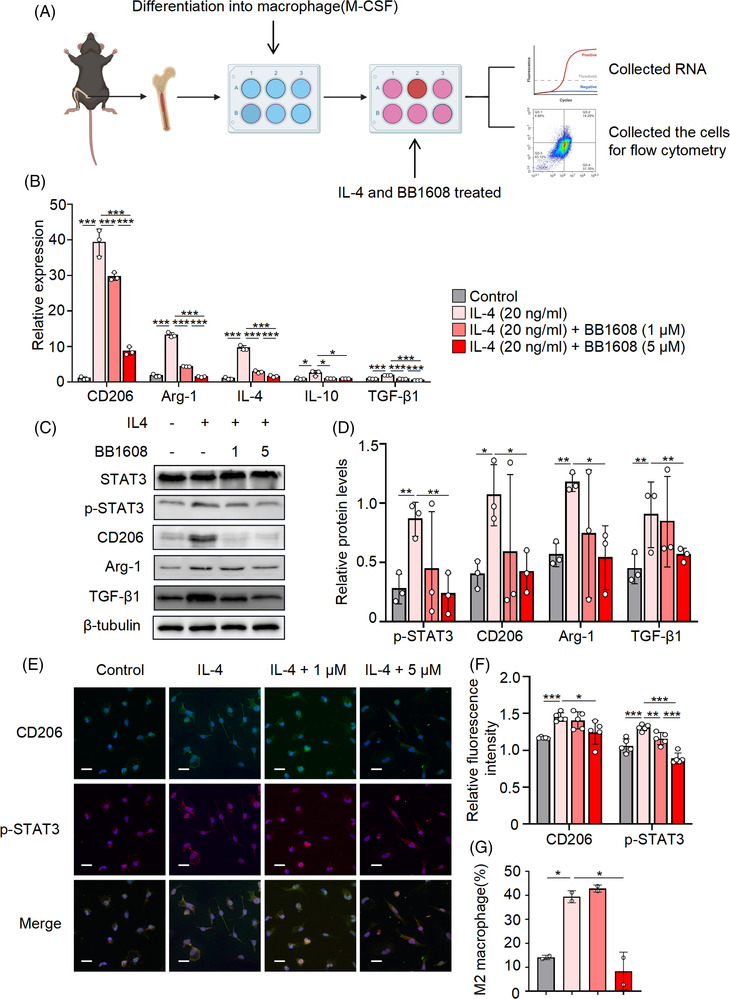
Phosphorylation of STAT3 acted as a regulator of M2 macrophage polarisation in vitro. BMDMs were extracted from the femurs of untreated wild‐type mice treated by M‐CSF and IL‐4 was added at 20 ng/ml in the culture medium. After cultured for 2 h, 5 and 1 μM of BB1608 (STAT3 inhibitor) was added into the medium for another 48 h and then the BMDMs were harvested for further analyses (A). The relative mRNA expressions of CD206, Arg‐1, IL‐4, IL‐10 and TGF‐β1 (B) in the BMDMs were quantified by qRT‐PCR. (C) The expression of total‐STAT3, the phosphorylation of STAT3, CD206, Arg‐1 and TGF‐β1 were determined by Western blot analysis on BMDM cells with indicated treatment. β‐Tubulin were used as a loading control. (D) Histograms showing the densitometry analysis of Western protein bands changes in the expression of phosphorylation of STAT3, CD206, Arg‐1 and TGF‐β1. (E) The BMDM cells were stained for p‐STAT3 (red) and CD206 (green) foci. DAPI staining (blue fluorescence), scale bar = 20 μm (magnification, 1000×). (F) Quantitative analysis of immunofluorescence staining for p‐STAT3 (red) and CD206 (green). (G) The proportion of M2 macrophage (CD45^+^ CD11b^+^ F4/80^+^ CD206^+^) was analysed by flow cytometry. Data were presented as mean ± SEM of three separated experiments, ^*^ *p* < .05, ^**^ *p* < .01, ^***^ *p* < .001

In prior study, we identified that M2a macrophages could promote the fibroblast secret profibrotic markers and ECM deposition. Then, we applied IL‐4 to stimulate macrophages to M2a phenotype. BMDMs were first treated with IL‐4 for 2 h and then with STAT3 inhibitors for 48 h. The samples were then collected for further analyses (Figure 6A). The M2‐related markers Arg‐1 and CD206 were upregulated after IL‐4 treatment and downregulated after STAT3 inhibitor treatment at 10 or 1 μM (Figure 6B). The phosphorylation of STAT3 and the expression levels of IL‐4, IL‐10 and TGF‐β1 were upregulated after IL‐4 treatment (Figure 6C,D). In Western blot and immunofluorescence, the results demonstrated that M2a macrophages polarisation is accompanied with the phosphorylation of STAT3 (Figure 6C–F). The proportion of M2 macrophages was reduced upon treatment with the STAT3 inhibitor (Figure 6G). The results suggested that polarisation of M2a macrophages regulated by STAT3 phosphorylation, in other words phosphorylation of STAT3 is required during the polarisation of M2a macrophages.

### 3.18 ROCK regulated the polarisation of M2 macrophages through STAT3 phosphorylation

**FIGURE 7:**
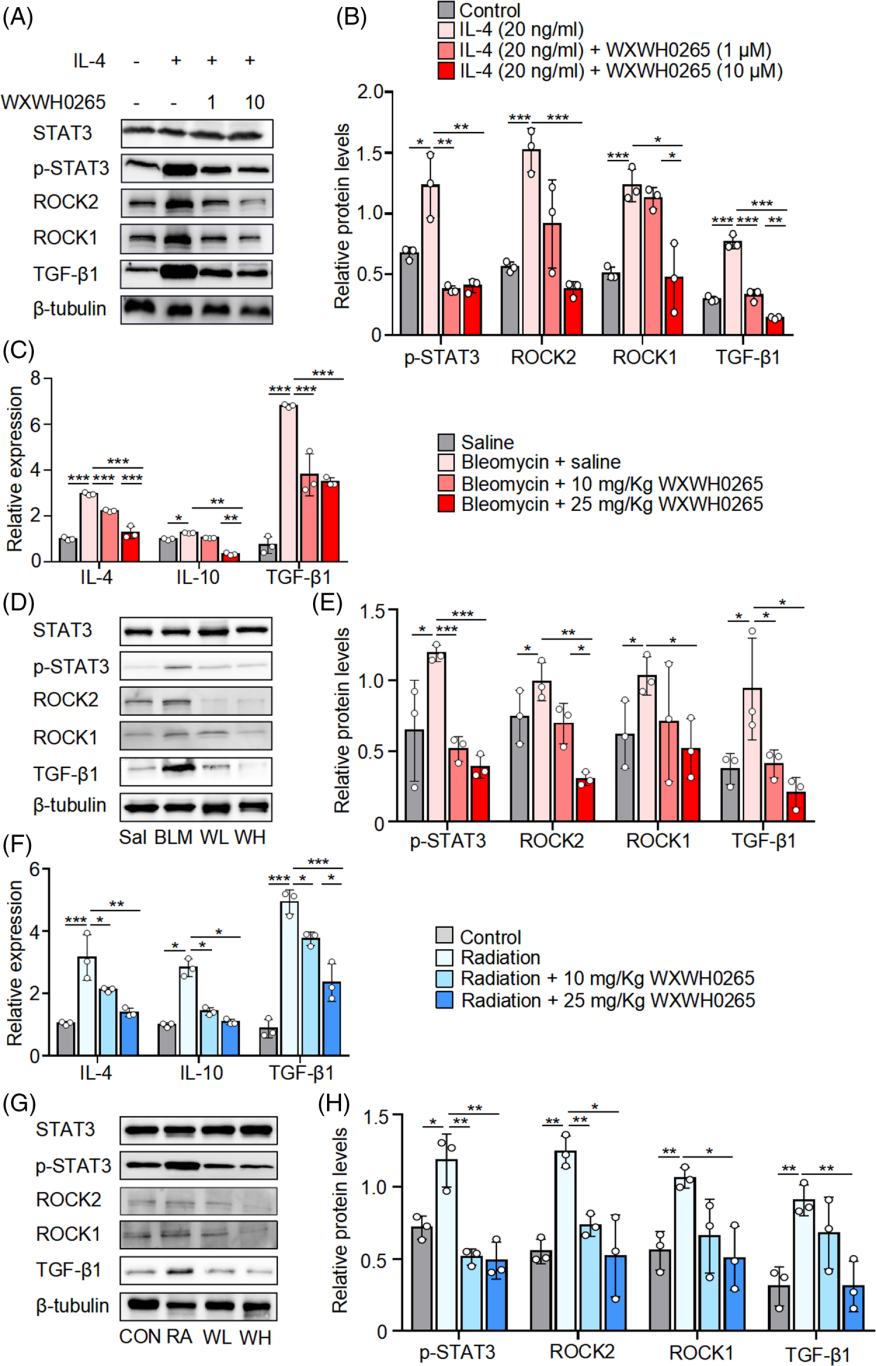
Reduced ROCK directs the M2 macrophages via downregulated STAT3 phosphorylation. BMDMs were extracted from the femurs of untreated wild‐type mice treated by M‐CSF and IL‐4 was added at 20 ng/ml in the culture medium. After cultured for 2 h, 10 and 1 μM of WXWH0265 was added into the medium for another 48 h and then the BMDMs were harvested for further analyses. Western blot analysis of the expression of ROCK1, ROCK2, p‐STAT3, STAT3 and TGF‐β1 in BMDMs treated with 20 ng/ml IL‐4 or the ROCK inhibitor WXWH0265 10 or 1 μM for 48 h (A). (B) Histograms showing the densitometry analysis of Western protein bands changes in the expression of ROCK1, ROCK2, p‐STAT3 and TGF‐β1. BMDMs were stimulated with IL‐4, LPS plus IgG and IL‐10, respectively. After cultured for 2 h, 10 and 1 μM of WXWH0265 was added into the medium for another 48 h and then the BMDMs were harvested for further analyses. After intratracheal instillation of bleomycin, mice were treated with WXWH0265 and scarified on day 28. The lung tissues were collected and digested for further analyses. (C) The relative mRNA expression of IL‐4, IL‐10 and TGF‐β1 in the lung on day 28. (D) Western blot analysis of the expression of ROCK1, ROCK2, p‐STAT3, STAT3 in lung tissue of bleomycin‐induced mice on day 28. β‐Tubulin were used as a loading control. BLM: bleomycin + saline; Sal: saline; WH: bleomycin + 25 mg/kg WXWH0265; WL: bleomycin + 10 mg/kg WXWH0265. (E) Histograms showing the densitometry analysis of Western protein bands changes in the expression of ROCK1, ROCK2, p‐STAT3 and TGF‐β1. (F) The relative mRNA expression of IL‐4, IL‐10 and TGF‐β1 in the lung on week 16. (G) Western blot analysis of the expression of ROCK1, ROCK2, p‐STAT3, STAT3 in lung tissue of radiation‐induced mice on week 16. β‐Tubulin were used as a loading control. Con: control; RA: radiation; WH: radiation + 25 mg/kg WXWH0265; WL: radiation + 10 mg/kg WXWH0265. (H) Histograms showing the densitometry analysis of Western protein bands changes in the expression of ROCK1, ROCK2, p‐STAT3 and TGF‐β1.Data were presented as mean ± SEM of three separated experiments *p < .05, ^**^ *p* < .01, ^***^ *p* < .001

According to results of previous experiments, M2 macrophages of IMs in lung play a leading role in the process of PF. The polarisation of M2 macrophages were significantly inhibited after WXWH0265 treatment (Figure 4D). To identify ROCK regulating the polarisation of M2 macrophages through STAT3 phosphorylation, the production of STAT3, p‐STAT3, ROCK1, ROCK2 and TGF‐β1 is further tested after M2 macrophages treated with WXWH0265 in vitro. Production levels of total STAT3, p‐STAT3, ROCK1, ROCK2 and TGF‐β1 were analysed by Western blot after IL‐4 or WXWH0265 treatment (Figure 7A,B). The phosphorylation of STAT3 were reduced after WXWH0265 treatment. Overall, the results showed that ROCK could inhibit M2a macrophages polarisation via regulating STAT3 phosphorylation.

**FIGURE 8:**
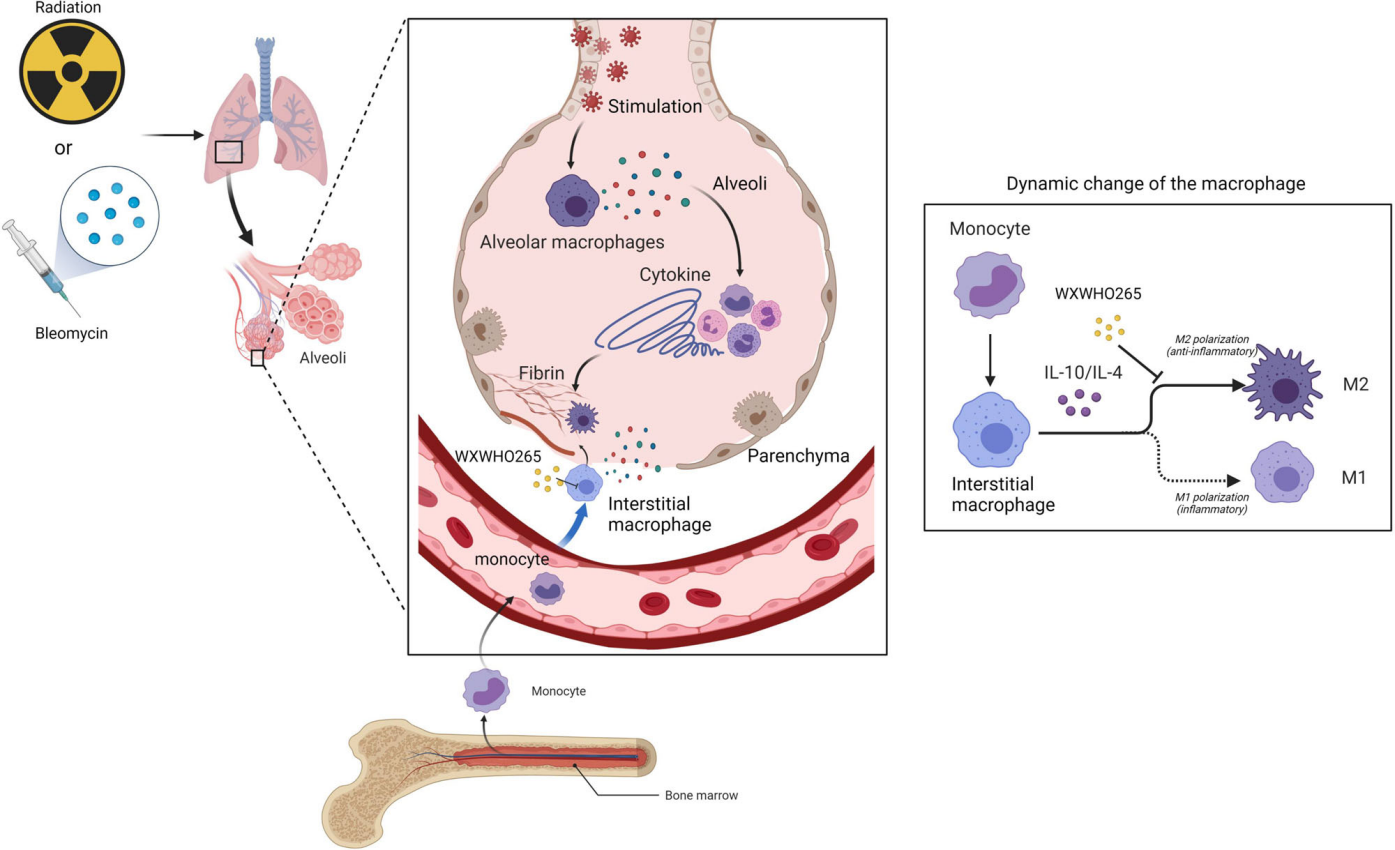
Elementary diagram of the proposed model of macrophages in lung contribution to radiation or bleomycin‐induced pulmonary fibrosis. After radiation or bleomycin stimulation, an increase of monocytes was observed in blood and lung. Several days later, the number of interstitial macrophages (pro‐inflammatory tissue‐infiltrating macrophages) was increased. In the interstitial space (parenchyma), secretion of cytokines such as interleukin‐4 (IL‐4) and IL‐10 is related to the number and the proportion of M2 macrophages derived from interstitial macrophages. The interplay between interstitial macrophages and fibroblasts contributes to pathogenesis of pulmonary fibrosis. WXWH0265, an unselective inhibitor of Rho‐associated coiled‐coil kinases (ROCK), could significantly inhibit the polarisation of M2 macrophages in parenchyma. Inhibiting the polarisation of M2 macrophage derived from interstitial macrophages, the fibrosis score and related markers are reduced

To identify the possible pathway by which ROCK may regulate PF in vivo, we assessed whether WXWH0265 regulates the polarisation of M2 macrophages via the phosphorylation of STAT3 in vivo in lung tissue. TGF‐β1 is a key element in assessing PF; therefore, we further tested the production of TGF‐β1 in vivo. In qRT‐PCR analysis, the expression levels of IL‐4, IL‐10 and TGF‐β1 were upregulated after bleomycin treatment on day 28 (Figure 7C). A higher dose of ROCK inhibitor correlated with a more significant reduction in the levels of IL‐4, IL‐10 and TGF‐β1 mRNA. We observed that STAT3 phosphorylation was significantly elevated in the bleomycin‐induced PF group, and unselective ROCK inhibitor‐treated group exhibited a significant reduction in STAT3 phosphorylation (Figure 7D,E). Similarly, the production of ROCK1, ROCK2 and TGF‐β1 proteins was regulated by bleomycin treatment and decreased by WXWH0265 treatment. In radiation‐induced PF, the expression levels of IL‐4, IL‐10 and TGF‐β1 were upregulated after radiation at week 16 (Figure 7F). WXWH0265 treatment substantially affected the expression of IL‐4 and IL‐10 in the lungs compared to that in radiation‐treated mice. We found that STAT3 phosphorylation was enhanced in response to radiation (Figure 7G,H). The whole process is described in Figure 8. ROCK inhibitors decreased ROCK1, ROCK2 and TGF‐β1 protein levels in a dose‐dependent manner. Higher doses of WXWH0265 significantly downregulated the secretion of these proteins. Taken together, these results support the activity of ROCK as a positive regulator of M2 macrophage polarisation both ex vivo and in vivo.

## 4 DISCUSSION

PF is a consequence of chronic lung injury and is associated with increased myofibroblasts, ECM deposition and alveolar structure damage.^63^ However, the pathological mechanism of PF at the molecular level remains largely unknown, which has substantially hampered the development of effective therapies against PF.^64^ In the present study, it was demonstrated that the unselective ROCK inhibitor WXWH0265 effectively alleviates bleomycin‐ and radiation‐induced PF. We identified that inhibition ROCK could inhibit the polarisation of M2 macrophages derived from BM in vivo and vitro. We provide evidence for profibrotic effect of M2 macrophages stimulated by IL‐4 in fibroblast activation and PF development. Collectively, inhibition ROCK to regulating polarisation of M2 macrophages is therapeutically correlated in PF development.

Prior studies have revealed that bleomycin and radiation can induce the activation of RhoA/ROCK signalling pathway in lung tissue.^28^ Although ROCK1 and ROCK2 share approximately 90% homology in their kinase domains and share many similar substrates, the cellular functions of these two isozymes are quite different.^28^ Considering that these two isozymes may affect the pathological process of PF through different mechanisms, an unselective ROCK inhibitor remains a superior option for the treatment of PF. Previous ROCK inhibitors have shown promising effects in PF treatment. The anti‐PF effects of various ROCK inhibitors, such as Y27632 and fasudil, have been tested.^65^, ^66^ However, the further use of these drugs is substantially limited due to their adverse effects.^67^ Symptomatic arterial hypotension was occasionally induced when previous ROCK inhibitors reached systemic circulation.^68^, ^69^ Non‐selective ROCK inhibitors (mainly Y27632) have been reported to have anti‐inflammatory actions caused by off‐target effects rather than by ROCK inhibition.^70^ Fasudil was the first and only ROCK inhibitor used to treat cerebral vasospasms following subarachnoid haemorrhage rather than PF in humans.^18^ In addition, fasudil was further tested and it demonstrated a prominent effect in oculopathy, angiocardiopathy and pulmonary diseases. WXWH0265 is a new type of unselective ROCK inhibitor developed based on previous agents. The histology in PF was more preserved with 25 mg/kg WXWH0265 than with other treatments, both macroscopically and microscopically. No significant toxic effects were observed. Further tests on WXWH0265 are required.

It has been reported that ROCK inhibitors could reduce the number of macrophages in the PF immune microenvironment.^18^ In breast cancer, it has been demonstrated that the polarisation of M2 macrophage was modulated by KLF14 via SOCS3/RhoA/ROCK signalling pathway, thereby suppressing tumour growth.^71^ In addition, inhibition of RhoA/ROCK signalling pathway suppresses macrophage polarisation.^72^ Our study illustrated that the ROCK inhibitor WXWH0265 inhibited M2 macrophage polarisation to reduce PF.

The infiltration of inflammatory cells increased in air spaces and lung interstitium after intratracheal instillation of bleomycin.^73^ The expression of cytokines, chemokines, reactive oxygen species and proteases in PF resulted in myofibroblast proliferation and collagen deposition.^74^ Monocytes, especially macrophages, play an important role in the process of PF. M1 (classically activated macrophages) and M2 (alternatively activated macrophages) are macrophage subtypes. M1 macrophages play an essential role in tissue inflammation.^75^ M2 macrophages are responsible for relieving tissue repair and ECM deposition.^76^ Macrophage polarisation is a dynamic process whereby macrophages maintain complex and functional phenotypes through the inflammation and fibrosis phases. M1 macrophages are responsible for wound healing after alveolar epithelial injury, while M2 macrophages are designated to resolve wound healing processes or terminate inflammatory responses in the lung.^77^ In patients with PF, the production of M2 markers is significantly increased. The number of M2 macrophages significantly increased in the late stage of PF. M2 macrophages can produce profibrotic mediators, such as TGF‐β1 and Connective tissue growth factor (CTGF) to cause violent and continuous fibroblast activation and facilitate myofibroblast proliferation.^43^ In our study, when M1 and M2 macrophages were depleted with clodronate liposomes, PF was significantly attenuated. WXWH0265 had no therapeutic effect in M2 macrophage‐depleted mice. These results support that ROCK inhibitor WXWH0265 eliminated PF through M2 macrophages.

Lung macrophages can be divided into AMs and IMs based on their location and source.^78^, ^79^ AMs usually reside in alveoli during the embryonic period and can self‐renew rather than transform into monocytes in peripheral blood system.^80^ Some studies have reported that AMs can also originate from monocytes in the peripheral circulation. It is usually considered that recruitment of AMs in lung is mainly associated with inflammatory phase, immune signalling and arginine metabolism, whereas AMs residing in lung mainly participates in proliferation and fatty acid metabolism.^81^, ^82^ The deletion of tissue‐resident AMs did not affect the severity of PF, while deleting monocyte‐derived AMs can attenuate PF, the effect remains debatable.^81^ However, the role of IMs in the pathology of PF has not been studied widely. When ly6C^high^ monocytes are stimulated by specific cytokines, the cells infiltrate lung tissue and transfer into IMs, which have been found to induce PF following repetitive administration of diphtheria toxin.^83^, ^84^ Some researches have revealed that IMs may also be derived from yolk sac macrophages.^85^ IMs produce high levels of IL‐10.^85^, ^86^ CD11c is a specific surface marker expressed on AMs.^79^, ^87^ In our study, the relative expression of M2‐related markers was downregulated after ROCK inhibitor treatment. Furthermore, we found that CD45^+^ F4/80^+^ CD206^+^ CD11c^–^ cells increased after stimulation with bleomycin or radiation, and decreased after ROCK inhibitor treatment. M1 and M2 macrophages were depleted by clodronate liposomes, but AMs were not significantly reduced. WXWH0265 had no inhibitory effect. Based on previous studies and our present results, we consider these cells to be M2 macrophages derived from IMs, leading to PF, and their downregulation could ameliorate PF after ROCK inhibitor treatment.

ROCK regulating the phosphorylation of STAT3 to promote macrophage polarisation is not a new concept, and has previously been reported in chronic graft versus host disease, hepatocellular carcinoma and breast cancer.^88^, ^89^, ^90^ To investigate the mechanism by which ROCK regulates macrophage polarisation, we elevated the phosphorylation of STAT3 in bleomycin‐ and radiation‐induced PF cells treated with WXWH0265, a ROCK inhibitor. The phosphorylation of STAT3 was inhibited by the decrease in M2 macrophage polarisation after WXWH0265 treatment, which verified our hypothesis. In previous studies, IL‐4 and IL‐10 levels were discovered to be significantly upregulated in bleomycin‐induced PF mice.^91^ Phosphorylation of STAT3 contributes to the deposition of collagen and release of IL‐10 and IL‐4 in tumours. Th2 cytokines, IL‐4 and IL‐10, can promote the polarisation of M2 macrophages, which are correlated with the pathogenesis of PF.^92^ The production of IL‐4 and IL‐10 was analysed in vitro and identified in vivo. The results showed that ROCK regulates STAT3 phosphorylation to promote M2 macrophage polarisation via IL‐4 and IL‐10. Both IL‐4 and IL‐10 can promote PF, and stimulated macrophages are transformed into various phenotypes.

M2 macrophages can be further classified into M2a, M2b and M2c subtypes based on their stimulation response. The analysis of macrophage phenotypes is typically performed in vitro in most cases to maintain easy control of extracellular environment. These results demonstrate that M2a could significantly promote ECM production. M2a has been reported to be involved in tissue repair and wound healing, and its role was verified in our study. M2b and M2c macrophages were significantly upregulated after stimulation by bleomycin and radiation; however, they could not directly facilitate α‐SMA and collagen‐Ⅰ production in vitro. TGF‐β production was accelerated by the M2a, M2b and M2c subtypes. Their cooperation may contribute to the PF process, but this correlation requires further exploration.

One of the most intriguing aspects of our outcomes was that unselective ROCK inhibitor WXWH0265 significantly attenuated bleomycin‐ and radiation‐induced PF by inhibiting M2 macrophage polarisation. This is a first study specifically describing the anti‐fibrosis effect of ROCK inhibitor in radiation‐induced PF. Our data indicated that the polarisation of M2 macrophages was suppressed by ROCK inhibition via downregulation of STAT3, which resulted in the amelioration of PF.

## 5 CONCLUSIONS

In conclusion, inhibition of ROCK significantly ameliorated bleomycin‐ and radiation‐induced PF in mice by regulating the polarisation of M2 macrophages via phosphorylation of STAT3. ROCK inhibited downstream phosphorylation of STAT3 to reduce the production of IL‐4 and IL‐10. We identified a vital role for M2 macrophages in PF and emphasised the importance of IMs during the fibrosis phase. These results provide empirical evidence of the role of ROCK in PF, and the ROCK inhibitor WXWH0265 may be a promising drug for the treatment of PF.

## CONFLICT OF INTEREST

The authors declare they have no conflicts of interest.

## Supporting information

Figure S10. Assessment of M2 macrophages‐related genes expression in lung tissue of radiation treatment mice.

Figure S9. The changes of absolute numbers and proportions of M2, M1 and AMwere analysed in radiation‐induced lung fibrosis.

Figure S8. Blockade of Rho‐associated coiled‐coil kinases (ROCK) inhibited the M2 macrophages infiltration in radiation‐induced fibrotic mice.

Figure S7. Assessment of M2 macrophages‐related genes expression in lung tissue of bleomycin treatment mice.

Figure S6. Clodronate liposomes had no effect on AMs.

Figure S5. The changes of absolute numbers and proportions of M2, M1 and AM were analysed in bleomycin‐induced lung fibrosis.

Figure S4. Evaluation of liver and kidney function in different groups.

Figure S3. Evaluation of drug toxicity in different groups.

Figure S2. Effect of WXWH0265, fasudil, KD025, GSK429286A and pirfenidone on bleomycin‐induced lung fibrosis.

Figure S1. ROCK inhibitor (WXWH0265) ameliorated pulmonary fibrosis and decreased the expression of ROCK1, ROCK2, α‐SMA and collagen‐Ⅰ in lung tissue.

Table S1 The names and sequences of primers used in qRT‐PCRexperiment GENE.
