# Supplementary figures and images for: Inhibition of ROCK ameliorates pulmonary fibrosis by suppressing M2 macrophage polarisation through phosphorylation of STAT3
Source: Clin Transl Med. 2022 Sep 30;12(10):e1036. doi: 10.1002/ctm2.1036 (PMC9523675; doi:10.1002/ctm2.1036)

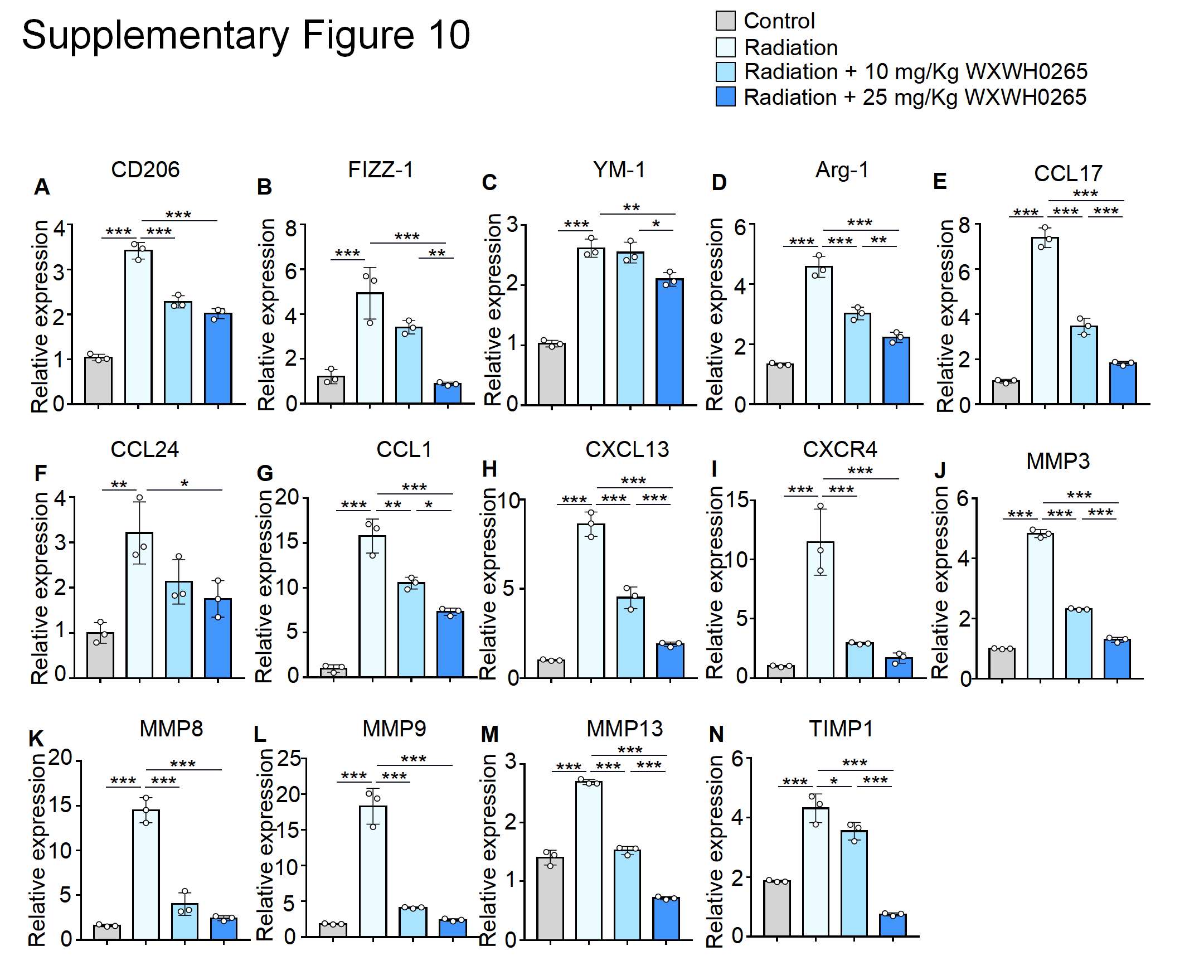

Supplement: Supplementary file 1 — Figure S10. Assessment of M2 macrophages‐related genes expression in lung tissue of radiation treatment mice. [file CTM2-12-e1036-s006.tif]

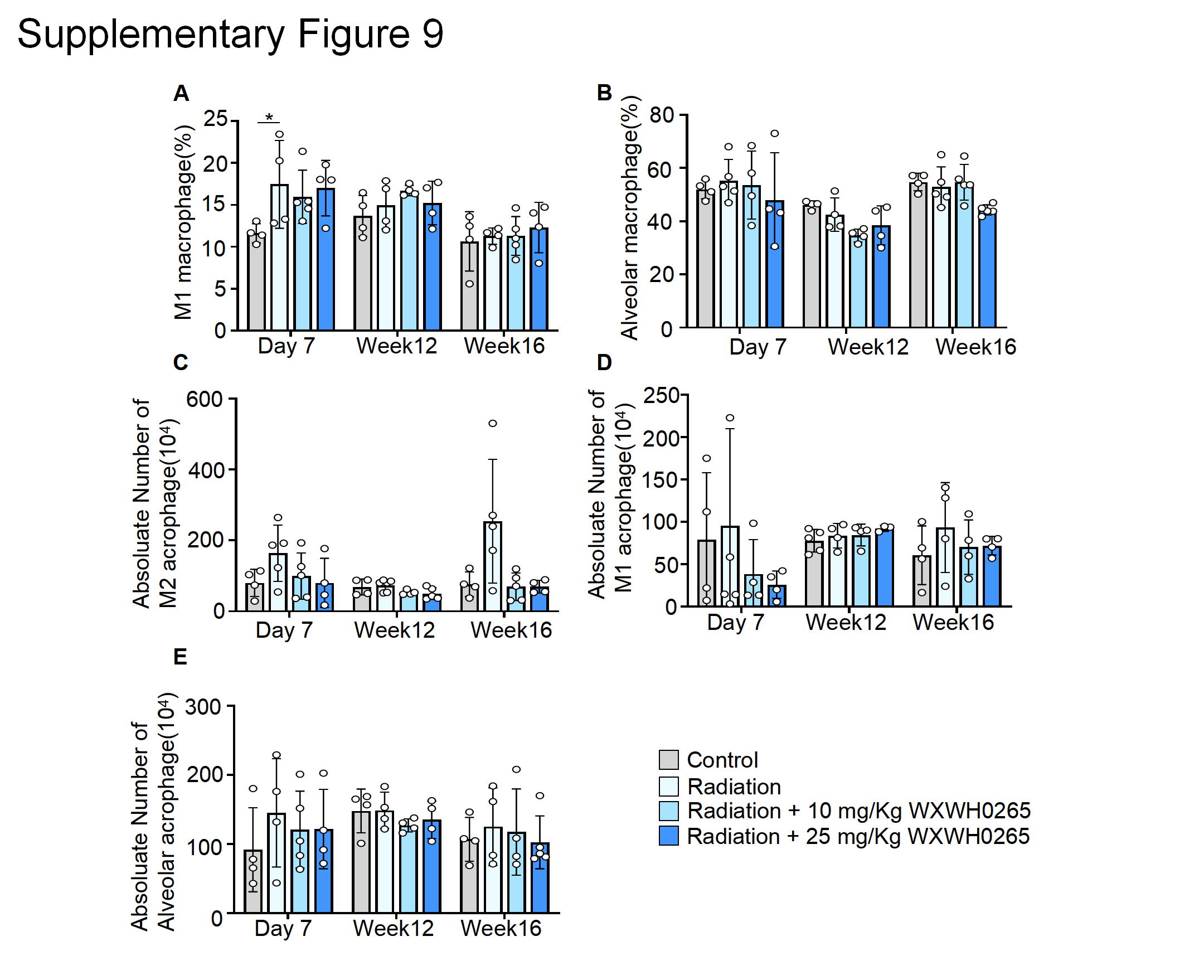

Supplement: Supplementary file 2 — Figure S9. The changes of absolute numbers and proportions of M2, M1 and AMwere analysed in radiation‐induced lung fibrosis. [file CTM2-12-e1036-s010.tif]

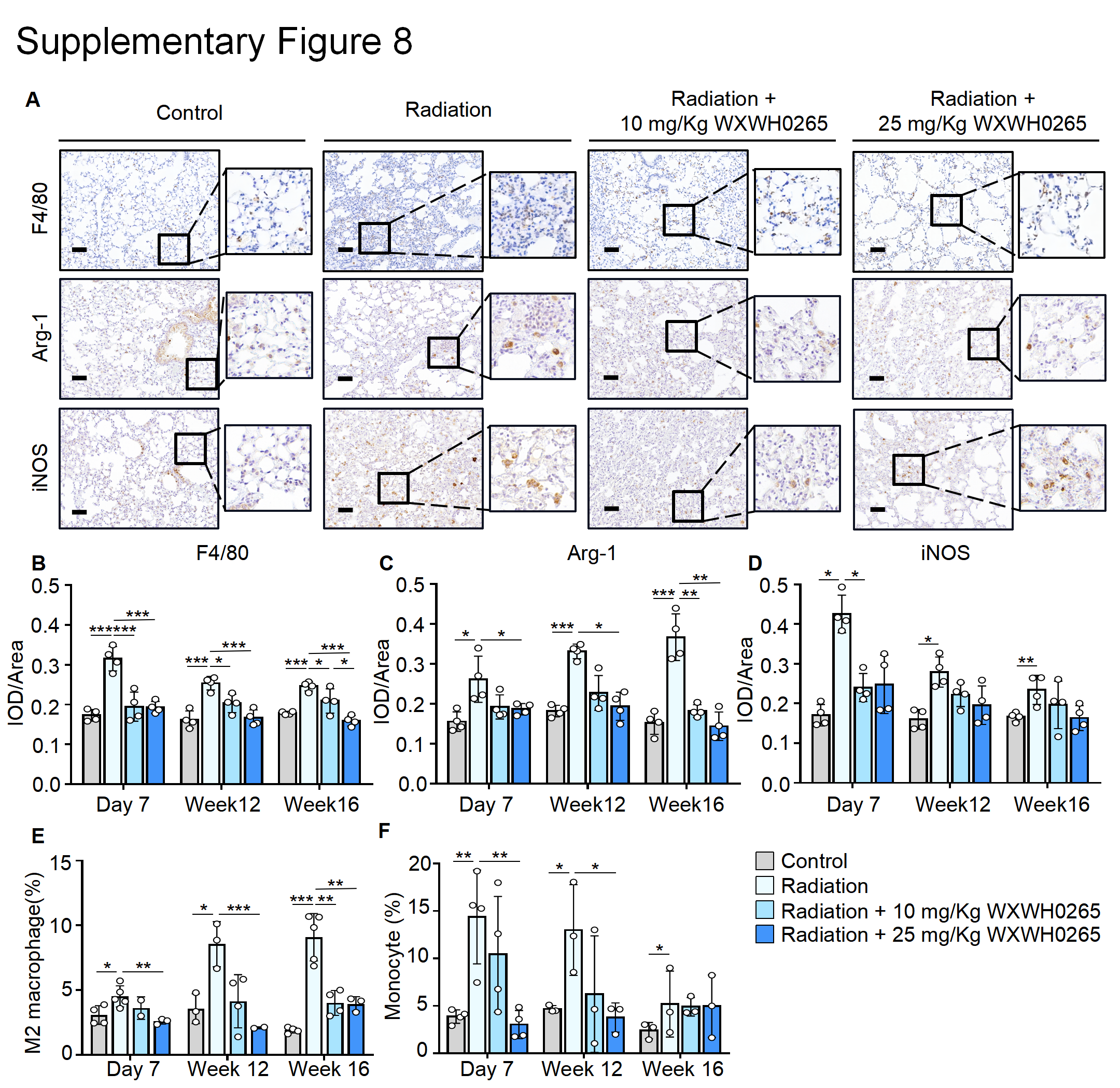

Supplement: Supplementary file 3 — Figure S8. Blockade of Rho‐associated coiled‐coil kinases (ROCK) inhibited the M2 macrophages infiltration in radiation‐induced fibrotic mice. [file CTM2-12-e1036-s011.tif]

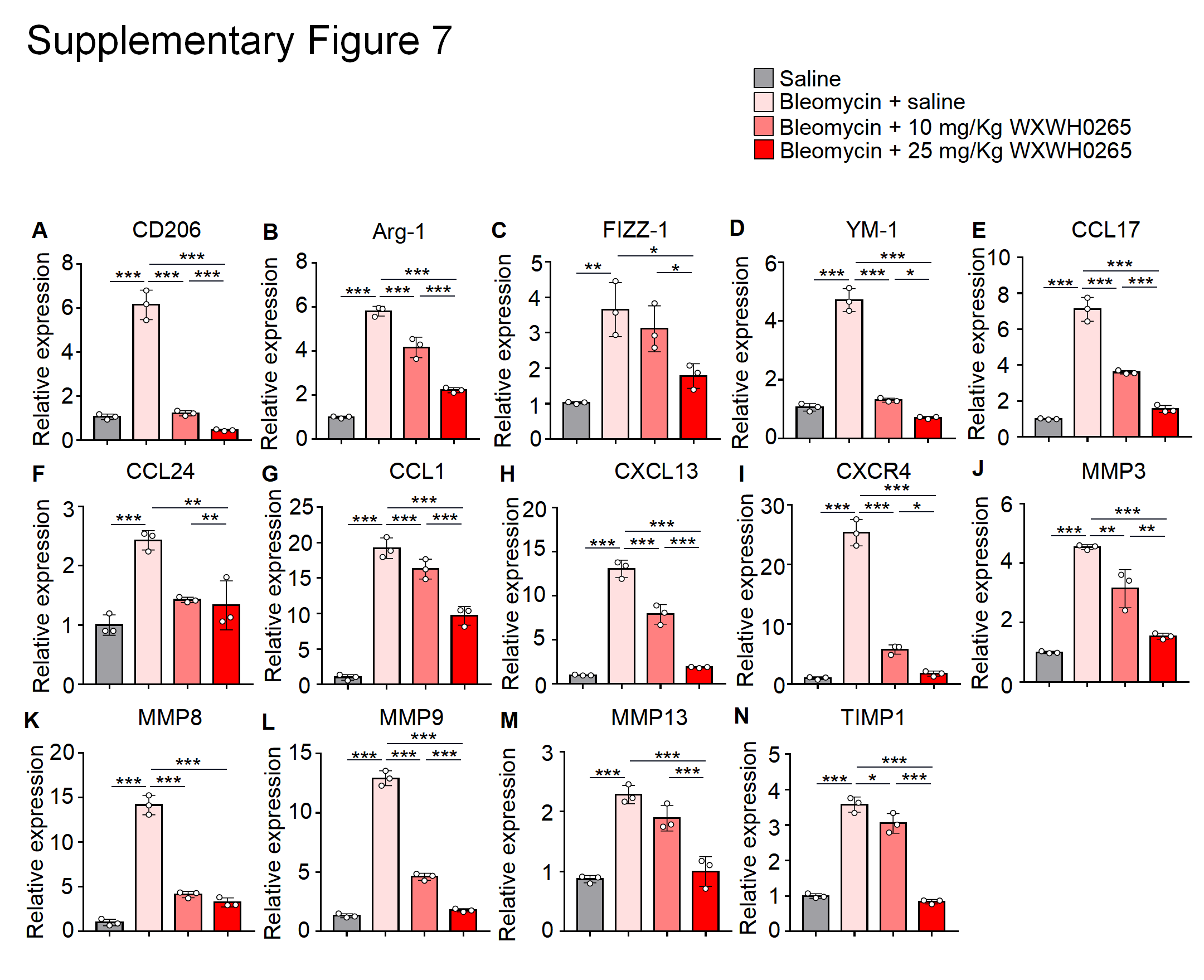

Supplement: Supplementary file 4 — Figure S7. Assessment of M2 macrophages‐related genes expression in lung tissue of bleomycin treatment mice. [file CTM2-12-e1036-s004.tif]

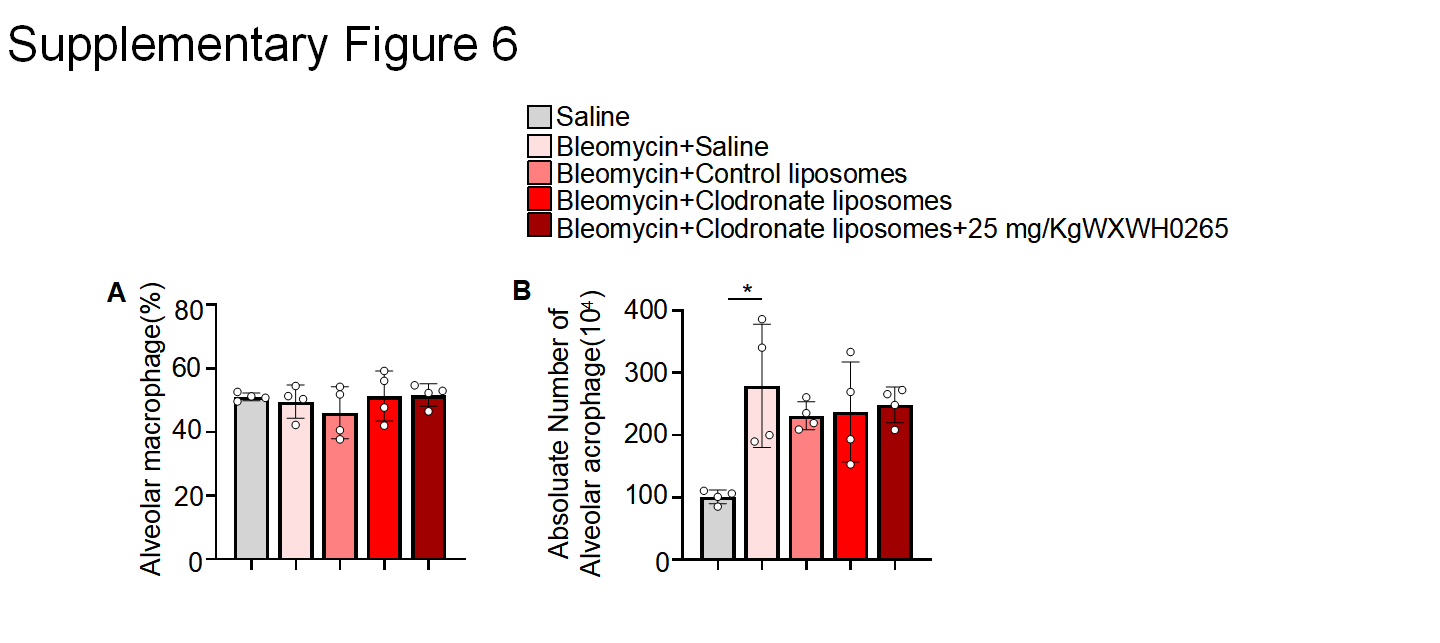

Supplement: Supplementary file 5 — Figure S6. Clodronate liposomes had no effect on AMs. [file CTM2-12-e1036-s005.tif]

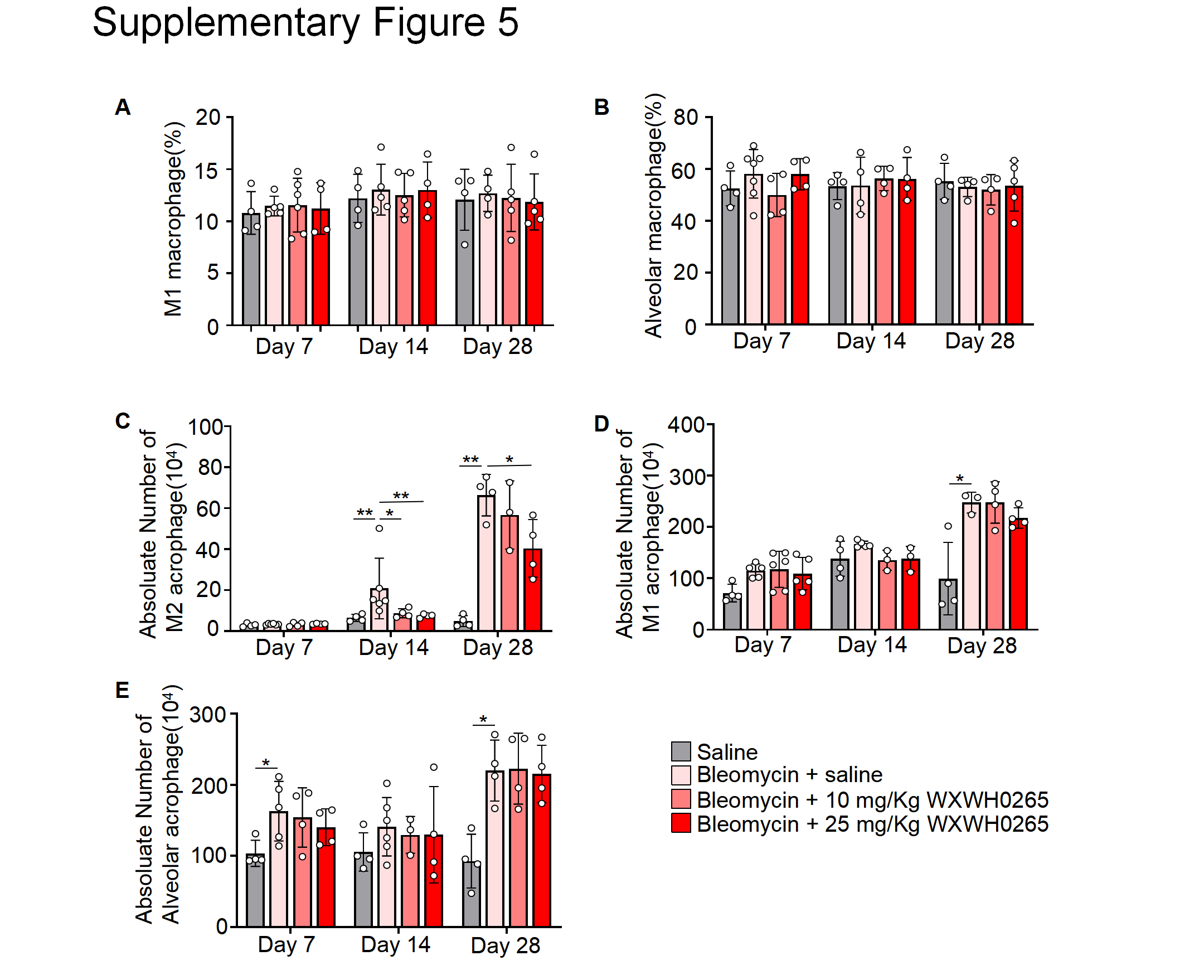

Supplement: Supplementary file 6 — Figure S5. The changes of absolute numbers and proportions of M2, M1 and AM were analysed in bleomycin‐induced lung fibrosis. [file CTM2-12-e1036-s001.tif]

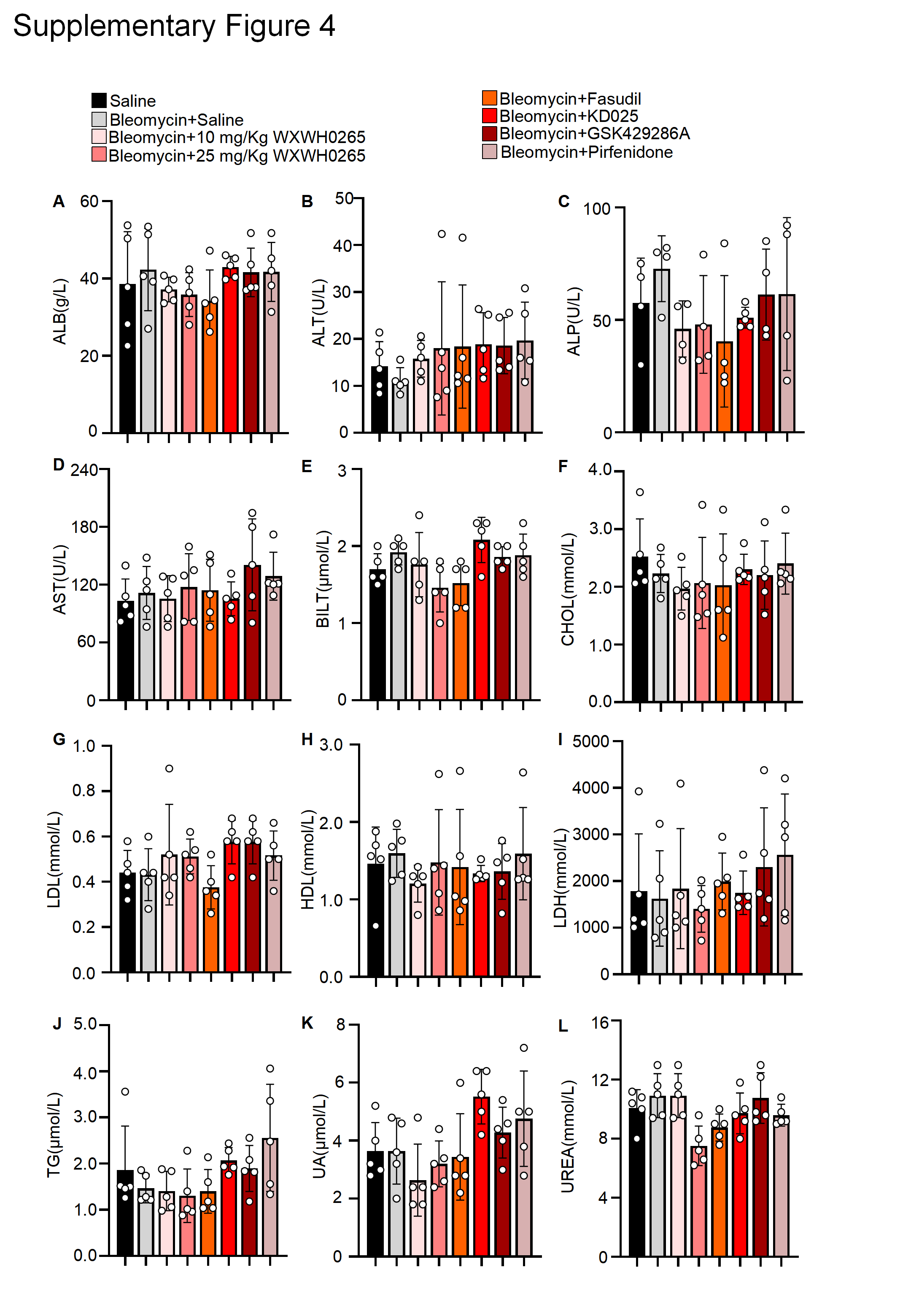

Supplement: Supplementary file 7 — Figure S4. Evaluation of liver and kidney function in different groups. [file CTM2-12-e1036-s003.tif]

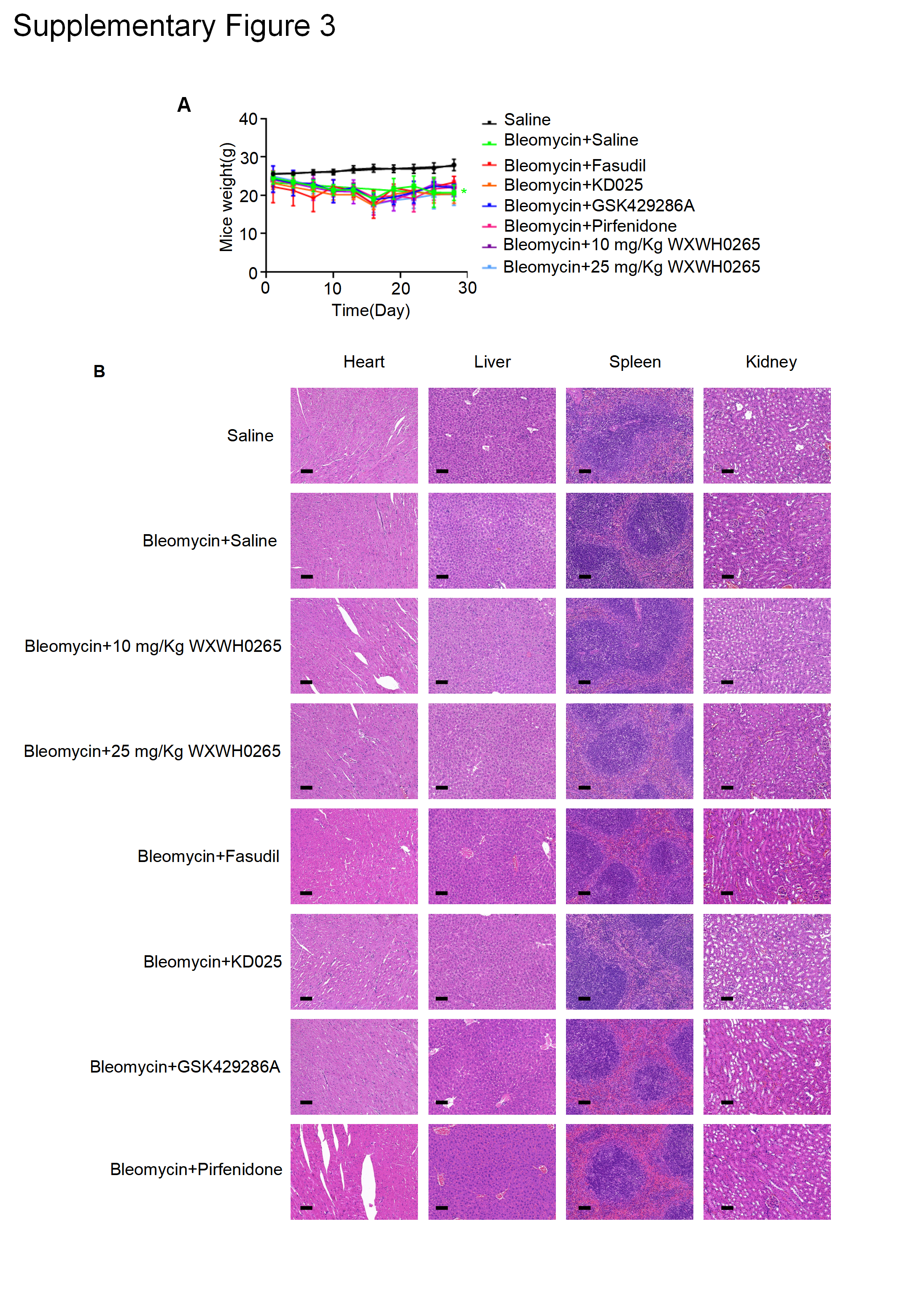

Supplement: Supplementary file 8 — Figure S3. Evaluation of drug toxicity in different groups. [file CTM2-12-e1036-s002.tif]

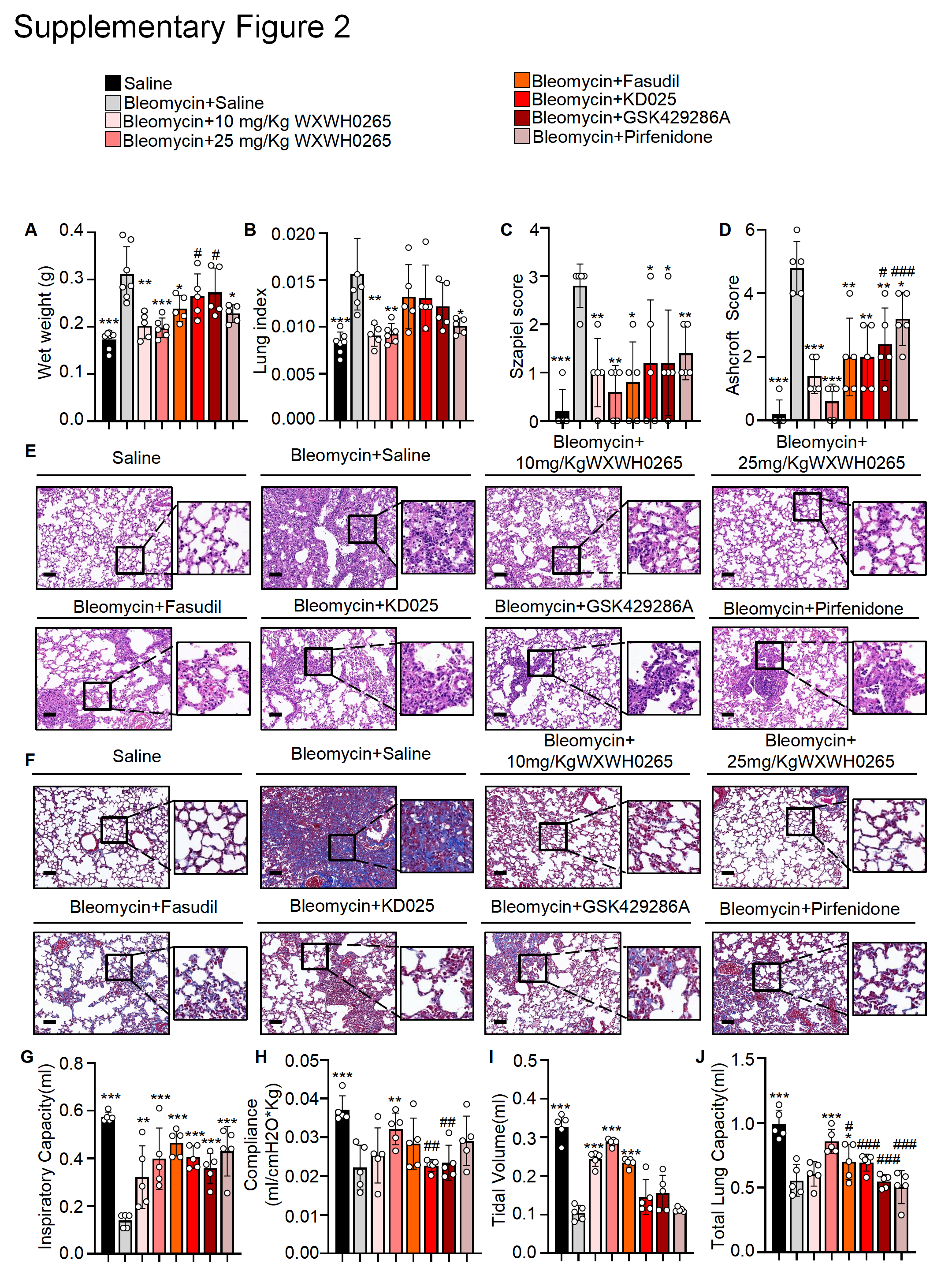

Supplement: Supplementary file 9 — Figure S2. Effect of WXWH0265, fasudil, KD025, GSK429286A and pirfenidone on bleomycin‐induced lung fibrosis. [file CTM2-12-e1036-s009.tif]

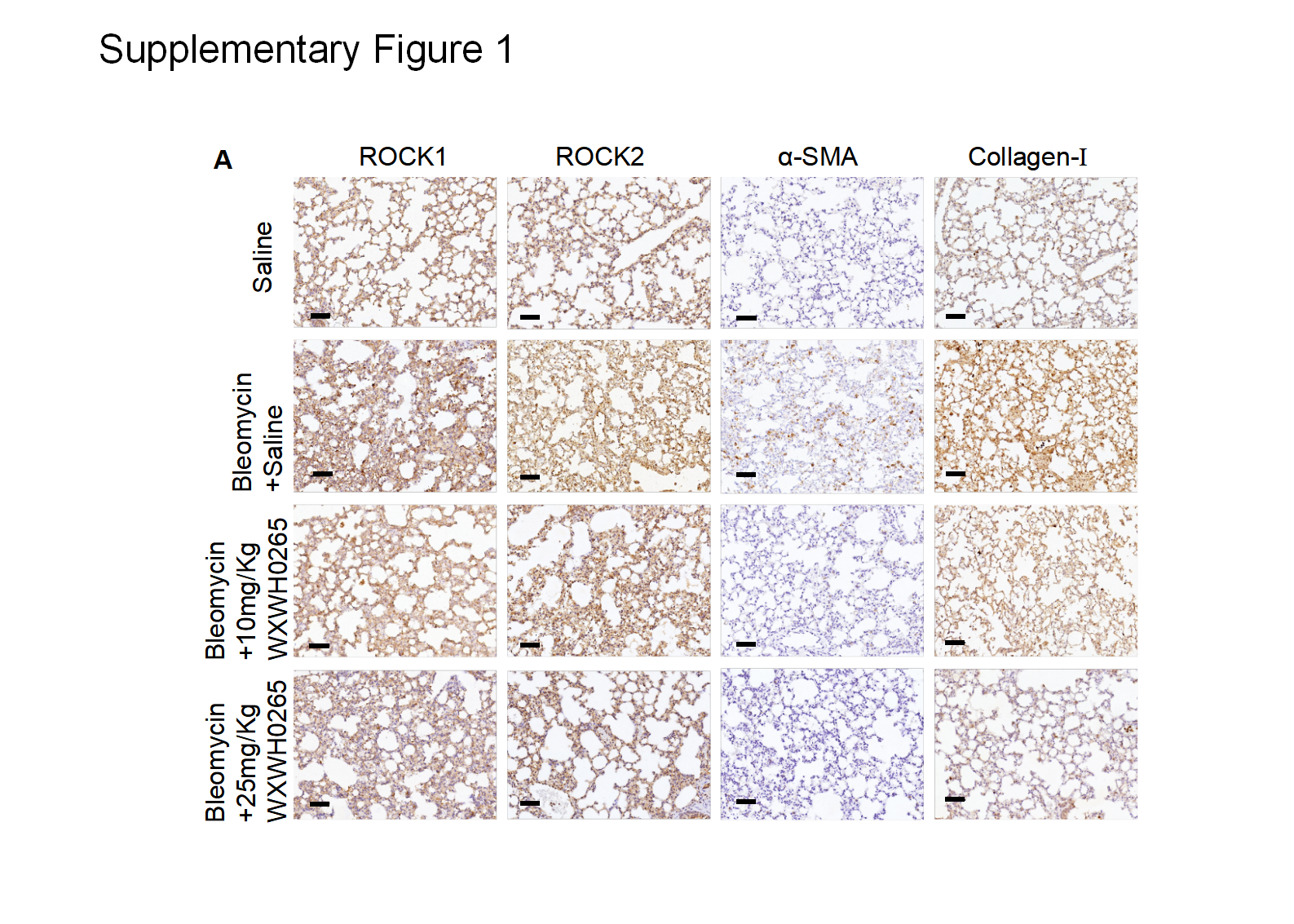

Supplement: Supplementary file 10 — Figure S1. ROCK inhibitor (WXWH0265) ameliorated pulmonary fibrosis and decreased the expression of ROCK1, ROCK2, α‐SMA and collagen‐Ⅰ in lung tissue. [file CTM2-12-e1036-s007.tif]
