# Supplementary material for: Inhibition of ROCK ameliorates pulmonary fibrosis by suppressing M2 macrophage polarisation through phosphorylation of STAT3
Source: Clin Transl Med. 2022 Sep 30;12(10):e1036. doi: 10.1002/ctm2.1036 (PMC9523675; doi:10.1002/ctm2.1036)
Supplement: Supplementary file 11 — Table S1 The names and sequences of primers used in qRT‐PCRexperiment GENE. [file CTM2-12-e1036-s008.docx]

**Supplementary Table 1 The names and sequences of primers used in PCR experiment GENE**

| Gene | Forward primer | Reverse primer |
| --- | --- | --- |
| GAPDH | 5’-CAACAGCAACTCCCACTCTTCCA-3’ | 5’-ACCCTGTTGCTGTAGCCGTAT-3’ |
| Arg-1 | 5’-CCTGAAGGAACTGAAAGGAAAG-3’ | 5’-TTGGCAGATATGCAGGGAGT-3’ |
| FIZZ-1 | 5’-CCCTCCACTGTAACGAAGACTC-3’ | 5’-CACACCCAGTAGCAGTCATCC-3’ |
| YM-1 | 5’-GAACACTGAGCTAAAAACTCTCCTG-3’ | 5’-GAGACCATGGCACTGAACG-3’ |
| CD206 | 5’-CTCTGTTCAGCTATTGGACGC-3’ | 5’-TGGCACTCCCAAACATAATTTGA-3’ |
| TGF-β1 | 5’-GACCGCAACAACGCCATCT-3’ | 5’-GCCCTGTATTCCGTCTCCTT-3’ |
| Collagen-1A1 | 5’-GCTCCTCTTAGGGGCCACT-3’ | 5’-CCACGTCTCACCATTGGGG-3’ |
| α-SMA | 5’-CTGCCGAGCGTGAGATTGT-3’ | 5’-CTTCGTCGTATTCCTGTTTGCT-3’ |
| CCL24 | 5’-TCTTGCTGCACGTCCTTTATT-3’ | 5’-GCATCCAGTTTTTGTATGTGCC-3’ |
| CCL17 | 5’-TACCATGAGGTCACTTCAGATGC-3’ | 5’-GCACTCTCGGCCTACATTGG-3’ |
| CCL1 | 5’-GGATGTTGACAGCAAGAGCA-3’ | 5’-ACAGGAGGAGCCCATCTTTC-3’ |
| CXCL13 | 5’-GGCCACGGTATTCTGGAAGC-3’ | 5’-ACCGACAACAGTTGAAATCACTC-3’ |
| CXCR4 | 5’-GAAGTGGGGTCTGGAGACTA-3’ | 5’-TTGCCGACTATGCCAGTCAAG-3’ |
| MMP3 | 5’-GGCCTGGAACAGTCTTGGC-3’ | 5’-TGTCCATCGTTCATCATCGTCA-3’ |
| MMP8 | 5’-TGCCACGATGGTTGCAGAG-3’ | 5’-AGGCATTTCCATAATCCCCATTG-3’ |
| MMP9 | 5’-GCAGAGGCATACTTGTACCG-3’ | 5’-TGATGTTATGATGGTCCCACTTG-3’ |
| MMP13 | 5’-TGTTTGCAGAGCACTACTTGAA-3’ | 5’-CAGTCACCTCTAAGCCAAAGAAA-3’ |
| TIMP-1 | 5’-CGAGACCACCTTATACCAGCG-3’ | 5’-ATGACTGGGGTGTAGGCGTA-3’ |
| IL-4 | 5’-GGTCTCAACCCCCAGCTAGT-3’ | 5’-GCCGATGATCTCTCTCAAGTGAT-3’ |
| IL-10 | 5’-GCTCTTACTGACTGGCATGAG-3’ | 5’-CGCAGCTCTAGGAGCATGTG-3’ |
